# Supplementary material for: A hierarchical framework for cortical and subcortical gray-matter parcellation across rodents, primates, and humans
Source: Nat Commun. 2026 Jul 23;17:8964. doi: 10.1038/s41467-026-75837-5 (PMC13503721; doi:10.1038/s41467-026-75837-5)
Supplement: Supplementary file 1 [file 41467_2026_75837_MOESM1_ESM.pdf]

Supplementary Table 1. Abbreviations used in the manuscript, organized by category.

Abbreviation	Full Term
Atlas sources	
ABA	Allen Brain Atlas (mouse)
BA	Brodmann areas (human)
BMA	Brain/MINDS Marmoset Reference Atlas
CIVM	Center for In Vivo Microscopy atlas (rhesus macaque)
FSS	FreeSurferSeg (human structural segmentation)
INM	INIA19–NeuroMaps atlas (rhesus macaque)
MBM	Marmoset Brain Mapping Atlas
Paxinos	Paxinos Histological Atlas (marmoset)
WHS	Waxholm Space atlas (rat)
Tissue classes (Level 0)	
GM	Gray Matter
WM	White Matter
DGM	Deep Gray Matter
CBL / CBGM	Cerebellum / Cerebellar Gray Matter
CSF	Cerebrospinal Fluid
Level-1 macro-regions	
FRO	Frontal Lobe
PAR	Parietal Lobe
TEM	Temporal Lobe
OCC	Occipital Lobe
INS	Insular Cortex
OLF	Olfactory Cortex
CIN	Cingulate Cortex
BG	Basal Ganglia
THL	Thalamic Areas
Level-2 subdivisions: Frontal	
PreC / FRO_Precentral	Precentral (primary motor) cortex
PreM / FRO_Premotor	Premotor (secondary motor) cortex
PrF / FRO_Prefrontal	Prefrontal cortex
Level-2 subdivisions: Parietal	
PAR	Parietal lobe (undivided in rodents)
PAR_Postcentral	Postcentral (primary somatosensory) cortex (primates)
PAR_Superior	Superior parietal lobule (primates)

PAR_Inferior	Inferior parietal lobule (primates)
PAR_Precuneus	Precuneus (primates)
Level-2 subdivisions: Temporal	
TemS / TEM_Superior	Superior temporal cortex
TemI / TEM_Inferior	Inferior temporal cortex
TemM / TEM_Medial	Medial temporal cortex (entorhinal/parahippocampal)
Hipp / TEM_Hippocampus	Hippocampus
Amyg / TEM_Amygdala	Amygdala
Level-2 subdivisions: Occipital	
OccL / OCC_Lateral	Lateral occipital cortex
OccM / OCC_Medial	Medial occipital cortex
Level-2 subdivisions: Insular	
InsA / INS_Anterior	Anterior insula
InsP / INS_Posterior	Posterior insula
Level-2 subdivisions: Olfactory	
OlfA / OLF_Anterior	Anterior olfactory cortex
Pir / OLF_Piriform	Piriform cortex
Level-2 subdivisions: Cingulate	
CingA / CIN_Anterior	Anterior cingulate cortex
CingP / CIN_Posterior	Posterior cingulate / retrosplenial cortex
Level-2 subdivisions: Basal Ganglia	
CPu / BG_CaudoPutamen	Caudoputamen (undivided in rodents; caudate + putamen in primates)
NAc / BG_Accumbens	Nucleus accumbens
Pall / BG_Pallidum	Globus pallidus
Level-2 subdivisions: Thalamic	
Thal / THL_Thalamus	Thalamus
Hypo / THL_Hypothalamus	Hypothalamus
Template and methodological terms	
MDT	Minimal Deformation Template
ICBM2009a	ICBM Nonlinear Asymmetric Human Template (2009a)
AC	Anterior commissure
AC–PC	Anterior commissure – Posterior commissure axis
CHA	Common Hierarchical Atlas
CDF	Cumulative Distribution Function
ROI	Region of Interest
U-Net	Convolutional neural network architecture used for tissue segmentation

SAMBA	Small Animal Multivariate Brain Analysis pipeline
ANTs	Advanced Normalization Tools
Imaging and acquisition terms	
dMRI	Diffusion magnetic resonance imaging
T1w / T2w	T1-weighted / T2-weighted MRI
GQI	Generalized q-sampling imaging
HARDI	High angular resolution diffusion imaging
DWI	Diffusion-weighted image
TR / TE	Repetition time / Echo time
Species abbreviations (used in Table 1)	
Mo	Mouse
Mz	Marmoset
Rh	Rhesus macaque
Hu / H	Human
Validation and statistical terms	
Dice	Dice similarity coefficient
R	Mean resultant length (geometric consistency)
FLNe	Fraction of extrinsic labeled neurons
Geo. R	Geometric consistency score (per-region mean of pairwise R)
Nom.	Nomenclature directness score

Supplementary Table 2. Spatial adjacencies of common hierarchical atlas regions across species.

Mouse

	FRO_Precentral	FRO_Premotor	FRO_Prefrontal	PAR	TEM_Superior	TEM_Inferior	TEM_Medial	TEM_Hippocampus	TEM_Amygdala	OCC_Lateral	OCC_Medial	INS_Anterior	INS_Posterior	OLF_Anterior	OLF_Piriform	CIN_Anterior	CIN_Posterior	BG_CaudoPutamen	BG_Accumbens	BG_Pallidum	THL_Thalamus	THL_Hypothalamus
FRO_Precentral		ADM		PL								AVL	PVL				PDM	PV				
FRO_Premotor	PVL		AVM									VL		AVM		PM	P					
FRO_Prefrontal		PDL										PVL		AV	PVL	PDM		PVL				
PAR	AM				PVL					P	PDM		VL				PDM	AVM				
TEM_Superior				ADM		V	PVM	M		PDM			AV					AVM				
TEM_Inferior					D		PM	DM	AVM				A									
TEM_Medial					ADL	AL		ADM	AVM	DM			A		AV							
TEM_Hippocampus					L	VL	PVL		AV	PDL	PD						DM	A			AVM	AVM
TEM_Amygdala						PDL	PDL	PD					DL	ADM	ADL			AD	ADM	D	PDM	PM
OCC_Lateral				A	AVL		VL	AVM			DM						ADM					
OCC_Medial				AVL				AV		VL							AM					
INS_Anterior	PDM	DM	ADM										PL		PVL			P				
INS_Posterior	ADM			DM	PD	P	P		VM			AM			AVM			ADM				
OLF_Anterior		PDL	PD						PVL						PVL				PV			
OLF_Piriform			ADM				PD		PVM			ADM	PDL	ADM								
CIN_Anterior		AL	AVL														PD					
CIN_Posterior	AVL	A		AVL				VL		PVL	PL					AV						
BG_CaudoPutamen	AD		ADM	PDL	PDL			P	PV			A	PVL						AVM	PV		
**BG_Accumbens**									PVL					AD				PDL				
**BG_Pallidum**									V									AD				VM
**THL_Thalamus**								PDL	AVL													
**THL_Hypothalamus**								PDL	AL											DL		

A: Anterior; P: Posterior; D: Dorsal; V: Ventral; M: Medial; L: Lateral

Marmoset

	FRO_Precentral	FRO_Premotor	FRO_Prefrontal	PAR	TEM_Superior	TEM_Inferior	TEM_Medial	TEM_Hippocampus	TEM_Amygdala	OCC_Lateral	OCC_Medial	INS_Anterior	INS_Posterior	OLF_Anterior	OLF_Piriform	CIN_Anterior	CIN_Posterior	BG_CaudoPutamen	BG_Accumbens	BG_Pallidum	THL_Thalamus	THL_Hypothalamus
FRO_Precentral		ADM	AVM	PD	PVL							PVL	PVL			AVM	PM					
FRO_Premotor	PVL		AV													AVM						
FRO_Prefrontal	PDL	PD			PL							PVL		PVL	PVL	PDM						
PAR	AV				VL					PV	PVM		AVL				VM					
TEM_Superior	ADM		AM	DM		PV				PM		AVM	AM									
TEM_Inferior					AD		PVM			PDM												
TEM_Medial						ADL		ADM	AM	PD	PDM											
TEM_Hippocampus							PVL		AV	PD	PDM						DM	AD			ADM	AM
TEM_Amygdala							PL	PD										D	ADM		PDM	DM
OCC_Lateral				AD	AL	AVL	AV	AV			ADM						ADM					
OCC_Medial				ADL			AVL	AVL		PVL							A					
INS_Anterior	ADM		ADM		PDL								PD		AVM							
INS_Posterior	ADM			PDM	PL							AV						M				
OLF_Anterior			ADM												PVL							
OLF_Piriform			ADM									PDL		ADM								
CIN_Anterior	PDL	PDL	AVL														PD					
CIN_Posterior	AL			DL				VL		PVL	P					AV						
BG_CaudoPutamen								PV	V				L						AVM	PVM	PM	
BG_Accumbens									PVL									PDL				PVM
BG_Pallidum																		ADL				
THL_Thalamus								PVL	AVL									AL				
THL_Hypothalamus								PL	VL										ADL			

A: Anterior; P: Posterior; D: Dorsal; V: Ventral; M: Medial; L: Lateral

Rhesus Macaque

	FRO_Precentral	FRO_Premotor	FRO_Prefrontal	PAR	TEM_Superior	TEM_Inferior	TEM_Medial	TEM_Hippocampus	TEM_Amygdala	OCC_Lateral	OCC_Medial	INS_Anterior	INS_Posterior	OLF_Anterior	OLF_Piriform	CIN_Anterior	CIN_Posterior	BG_CaudoPutamen	BG_Accumbens	BG_Pallidum	THL_Thalamus	THL_Hypothalamus
FRO_Precentral		ADM	AVM	P	PVL											AM	PM					
FRO_Premotor	PVL		AVL													AVM						
FRO_Prefrontal	PDL	PDM		PD								PVL		PVM	PV	PDM		P	PVM	PV		
PAR	A		AV		AVL					PV	PVM	AVL	AVL			AM	M					
TEM_Superior	ADM			PDM		PV	PVM		AVM	PDM		AM	DM		AVM							
TEM_Inferior					AD		PM	M		PDM												
TEM_Medial					ADL	AL		A	AM	PD	PDM				A						ADM	
TEM_Hippocampus						L	P		AVM		PDM						DM	ADM			DM	
TEM_Amygdala					PDL		PL	PDL							ADL			AD		D		DM
OCC_Lateral				AD	AVL	AVL	AV				AM											
OCC_Medial				ADL			AVL	AVL		PL							ADM					
INS_Anterior			ADM	PDM	PL								PD		AVM							
INS_Posterior				PDM	VL							AV										
OLF_Anterior			ADL												PVL				PDM			
OLF_Piriform			AD		PDL		P		PVM			PDL		ADM				PDM	DM			
CIN_Anterior	PL	PDL	AVL	PL													P					
CIN_Posterior	AL			L				VL			PVL					A						
BG_CaudoPutamen			A					PVL	PV						AVL				AVM	PVM	PM	
BG_Accumbens			ADL											AVL	VL			PDL		PDL		
BG_Pallidum			AD						V									ADL	AVM			VM
THL_Thalamus							PVL	VL										AL				AVM
THL_Hypothalamus									VL											DL	PDL	

A: Anterior; P: Posterior; D: Dorsal; V: Ventral; M: Medial; L: Lateral

Human

	FRO_Precentral	FRO_Premotor	FRO_Prefrontal	PAR	TEM_Superior	TEM_Inferior	TEM_Medial	TEM_Hippocampus	TEM_Amygdala	OCC_Lateral	OCC_Medial	INS_Anterior	INS_Posterior	OLF_Anterior	OLF_Piriform	CIN_Anterior	CIN_Posterior	BG_CaudoPutamen	BG_Accumbens	BG_Pallidum	THL_Thalamus	THL_Hypothalamus
FRO_Precentral		ADM	AVM	P								AV					PVM					
FRO_Premotor	PVL		AVL														PVM					
FRO_Prefrontal	PDL	PDM							PV			PVL		PVM	PV	VM	PDM	PVM	PVM			PVM
PAR	A				AVL	AVL				PV	PVM	AV	AV				AVM					
TEM_Superior				PDM		PVM	PVM		AVM	PDM		ADM	DM		AVM							
TEM_Inferior				PDM	ADL		M			PDM												
TEM_Medial					ADL	L		ADM	AM	PD	PDM				A		DM					
TEM_Hippocampus							PVL		AV		PDM						DM				DM	
TEM_Amygdala			AD		PDL		PL	PD							AL			AD		D		
OCC_Lateral				AD	AVL	AVL	AV				AVM											
OCC_Medial				ADL			AVL	AVL		PDL							AD					
INS_Anterior	PD		ADM	PD	PVL								PL		VM							
INS_Posterior				PD	VL							AM			AVM							
OLF_Anterior			ADL												PL							
OLF_Piriform			AD		PDL		P		PM			DL	PDL	AM								
CIN_Anterior			DL														PD	PL	PV			
CIN_Posterior	ADL	ADL	AVL	PDL			VL	VL			PV					AV						
BG_CaudoPutamen			ADL						PV							AM			AVM	PV	PM	
BG_Accumbens			ADL													AD		PDL				
BG_Pallidum									V									AD			PDM	
THL_Thalamus								VL										AL		AVL		
THL_Hypothalamus			ADL																			

A: Anterior; P: Posterior; D: Dorsal; V: Ventral; M: Medial; L: Lateral

Supplementary Table 3. Per-connection agreement between mouse and marmoset tracer data.

source	target	mouse	marmoset	marmoset_signal_min	marmoset_signal_mean	mouse_rank_pct	marmoset_rank_pct	agreement	coverage_quartile
FRO_Precentral	FRO_Premotor	0.910	0.571	6.078	6.409	0.842	0.842	1.000	Q3
FRO_Premotor	FRO_Prefrontal	0.668	0.438	6.740	12.060	0.792	0.782	0.990	Q4 (dense)
FRO_Prefrontal	FRO_Premotor	1.091	0.586	6.740	12.060	0.871	0.861	0.990	Q4 (dense)
OCC_Lateral	TEM_Superior	0.788	0.509	7.284	12.989	0.832	0.822	0.990	Q4 (dense)
CIN_Anterior	PAR	0.011	0.071	4.120	17.244	0.406	0.416	0.990	Q1 (sparse)
FRO_Precentral	OCC_Lateral	0.000	0.009	6.078	6.681	0.168	0.188	0.980	Q3
OCC_Medial	TEM_Inferior	0.002	0.022	4.359	4.801	0.267	0.287	0.980	Q2
FRO_Precentral	PAR	1.948	1.026	6.078	18.223	0.921	0.950	0.970	Q3
FRO_Precentral	TEM_Medial	0.000	0.001	1.312	3.695	0.030	0.059	0.970	Q1 (sparse)
FRO_Premotor	CIN_Posterior	0.041	0.129	5.338	6.039	0.545	0.515	0.970	Q3
PAR	FRO_Precentral	2.557	1.435	6.078	18.223	0.970	1.000	0.970	Q3
PAR	TEM_Inferior	0.014	0.110	4.359	17.364	0.436	0.465	0.970	Q2
CIN_Anterior	FRO_Prefrontal	1.222	0.949	4.120	10.750	0.891	0.921	0.970	Q1 (sparse)
CIN_Posterior	FRO_Premotor	0.051	0.183	5.338	6.039	0.564	0.594	0.970	Q3
OCC_Lateral	FRO_Precentral	0.000	0.008	6.078	6.681	0.129	0.168	0.960	Q3
OCC_Lateral	FRO_Premotor	0.002	0.011	6.740	7.012	0.257	0.218	0.960	Q4 (dense)
OCC_Lateral	FRO_Prefrontal	0.013	0.105	7.284	12.332	0.416	0.455	0.960	Q4 (dense)
FRO_Premotor	TEM_Superior	0.000	0.010	6.740	12.717	0.149	0.198	0.950	Q4 (dense)
CIN_Anterior	TEM_Medial	0.000	0.000	1.312	2.716	0.059	0.010	0.950	Q1 (sparse)
FRO_Prefrontal	CIN_Anterior	1.177	0.983	4.120	10.750	0.881	0.941	0.941	Q1 (sparse)
CIN_Anterior	FRO_Precentral	0.022	0.085	4.120	5.099	0.485	0.426	0.941	Q1 (sparse)
FRO_Premotor	FRO_Precentral	1.335	0.549	6.078	6.409	0.901	0.832	0.931	Q3
PAR	TEM_Superior	1.069	0.981	18.694	24.531	0.861	0.931	0.931	Q4 (dense)
PAR	OCC_Lateral	0.381	0.334	7.284	18.826	0.762	0.693	0.931	Q4 (dense)
FRO_Prefrontal	TEM_Medial	0.001	0.005	1.312	9.346	0.198	0.119	0.921	Q1 (sparse)
FRO_Prefrontal	OCC_Medial	0.003	0.045	5.244	11.312	0.277	0.356	0.921	Q2
OCC_Medial	FRO_Premotor	0.010	0.028	5.244	5.992	0.386	0.307	0.921	Q2
TEM_Medial	TEM_Inferior	0.356	0.291	1.312	2.835	0.752	0.663	0.911	Q1 (sparse)
OCC_Lateral	OCC_Medial	3.341	0.716	5.244	6.264	0.990	0.901	0.911	Q2
OCC_Medial	FRO_Precentral	0.000	0.008	5.244	5.661	0.089	0.178	0.911	Q2
OCC_Medial	FRO_Prefrontal	0.029	0.087	5.244	11.312	0.525	0.436	0.911	Q2

PAR	TEM_Medial	0.008	0.016	1.312	15.841	0.356	0.257	0.901	Q1 (sparse)
PAR	OCC_Medial	0.154	0.410	5.244	17.806	0.653	0.752	0.901	Q2
PAR	CIN_Anterior	0.021	0.164	4.120	17.244	0.465	0.564	0.901	Q1 (sparse)
CIN_Posterior	FRO_Precentral	0.004	0.063	5.338	5.708	0.297	0.396	0.901	Q3
CIN_Posterior	TEM_Inferior	0.001	0.017	4.359	4.848	0.178	0.277	0.901	Q2
TEM_Superior	OCC_Medial	0.120	0.136	5.244	11.969	0.634	0.525	0.891	Q2
TEM_Inferior	CIN_Posterior	0.000	0.006	4.359	4.848	0.040	0.149	0.891	Q2
TEM_Medial	CIN_Anterior	0.001	0.004	1.312	2.716	0.188	0.079	0.891	Q1 (sparse)
PAR	FRO_Premotor	0.955	1.037	6.740	18.554	0.851	0.970	0.881	Q4 (dense)
TEM_Medial	FRO_Prefrontal	0.025	0.062	1.312	9.346	0.505	0.386	0.881	Q1 (sparse)
OCC_Medial	TEM_Superior	0.137	0.414	5.244	11.969	0.644	0.762	0.881	Q2
OCC_Medial	OCC_Lateral	5.053	0.655	5.244	6.264	1.000	0.881	0.881	Q2
FRO_Precentral	TEM_Superior	0.009	0.015	6.078	12.386	0.376	0.248	0.871	Q3
FRO_Premotor	PAR	0.162	0.455	6.740	18.554	0.663	0.792	0.871	Q4 (dense)
PAR	FRO_Prefrontal	0.053	0.342	17.380	23.874	0.574	0.713	0.861	Q4 (dense)
TEM_Superior	OCC_Lateral	0.729	0.334	7.284	12.989	0.822	0.683	0.861	Q4 (dense)
TEM_Inferior	TEM_Superior	0.181	0.505	4.359	11.526	0.673	0.812	0.861	Q2
CIN_Anterior	TEM_Superior	0.000	0.007	4.120	11.407	0.020	0.158	0.861	Q1 (sparse)
TEM_Superior	TEM_Inferior	0.266	0.645	4.359	11.526	0.723	0.871	0.851	Q2
TEM_Superior	CIN_Posterior	0.001	0.057	5.338	12.016	0.228	0.376	0.851	Q3
FRO_Precentral	CIN_Anterior	0.056	0.369	4.120	5.099	0.584	0.743	0.842	Q1 (sparse)
TEM_Superior	PAR	0.712	1.051	18.694	24.531	0.812	0.980	0.832	Q4 (dense)
CIN_Posterior	CIN_Anterior	0.246	0.149	4.120	4.729	0.703	0.535	0.832	Q1 (sparse)
TEM_Inferior	OCC_Medial	0.000	0.013	4.359	4.801	0.050	0.228	0.822	Q2
TEM_Inferior	CIN_Anterior	0.000	0.027	4.120	4.239	0.119	0.297	0.822	Q1 (sparse)
CIN_Posterior	OCC_Medial	2.460	0.424	5.244	5.291	0.960	0.772	0.812	Q2
FRO_Premotor	TEM_Medial	0.001	0.000	1.312	4.026	0.238	0.040	0.802	Q1 (sparse)
CIN_Posterior	TEM_Superior	0.001	0.097	5.338	12.016	0.248	0.446	0.802	Q3
FRO_Precentral	CIN_Posterior	0.010	0.187	5.338	5.708	0.396	0.604	0.792	Q3
CIN_Anterior	FRO_Premotor	1.996	0.359	4.120	5.430	0.941	0.733	0.792	Q1 (sparse)
FRO_Precentral	FRO_Prefrontal	0.017	0.304	6.078	11.729	0.455	0.673	0.782	Q3
FRO_Precentral	OCC_Medial	0.003	0.002	5.244	5.661	0.287	0.069	0.782	Q2
FRO_Prefrontal	FRO_Precentral	0.013	0.283	6.078	11.729	0.426	0.653	0.772	Q3
FRO_Premotor	OCC_Medial	0.022	0.013	5.244	5.992	0.475	0.238	0.762	Q2
TEM_Inferior	TEM_Medial	0.321	0.123	1.312	2.835	0.743	0.495	0.752	Q1 (sparse)
TEM_Superior	FRO_Premotor	0.000	0.041	6.740	12.717	0.069	0.327	0.743	Q4 (dense)
TEM_Inferior	FRO_Premotor	0.000	0.017	4.359	5.549	0.010	0.267	0.743	Q2
CIN_Anterior	CIN_Posterior	0.105	0.052	4.120	4.729	0.624	0.366	0.743	Q1 (sparse)
TEM_Superior	CIN_Anterior	0.000	0.045	4.120	11.407	0.079	0.347	0.733	Q1 (sparse)
CIN_Anterior	OCC_Lateral	0.004	0.000	4.120	5.702	0.307	0.020	0.713	Q1 (sparse)
CIN_Posterior	TEM_Medial	0.006	0.000	1.312	3.325	0.317	0.030	0.713	Q1 (sparse)

OCC_Lateral	PAR	0.244	1.147	7.284	18.826	0.693	0.990	0.703	Q4 (dense)
OCC_Medial	PAR	0.072	0.825	5.244	17.806	0.614	0.911	0.703	Q2
OCC_Lateral	TEM_Inferior	0.006	0.266	4.359	5.821	0.327	0.634	0.693	Q2
OCC_Lateral	CIN_Posterior	0.259	0.065	5.338	6.311	0.713	0.406	0.693	Q3
TEM_Medial	TEM_Superior	0.678	0.116	1.312	10.003	0.802	0.485	0.683	Q1 (sparse)
FRO_Prefrontal	CIN_Posterior	0.001	0.161	5.338	11.359	0.218	0.545	0.673	Q3
CIN_Posterior	PAR	0.049	0.682	5.338	17.854	0.554	0.891	0.663	Q3
FRO_Premotor	CIN_Anterior	1.953	0.183	4.120	5.430	0.931	0.584	0.653	Q1 (sparse)
PAR	CIN_Posterior	0.059	1.029	5.338	17.854	0.604	0.960	0.644	Q3
OCC_Lateral	CIN_Anterior	0.015	0.004	4.120	5.702	0.446	0.089	0.644	Q1 (sparse)
FRO_Premotor	OCC_Lateral	0.023	0.006	6.740	7.012	0.495	0.129	0.634	Q4 (dense)
TEM_Medial	OCC_Medial	0.217	0.038	1.312	3.278	0.683	0.317	0.634	Q1 (sparse)
TEM_Inferior	FRO_Prefrontal	0.008	0.352	4.359	10.869	0.347	0.723	0.624	Q2
TEM_Medial	OCC_Lateral	1.600	0.124	1.312	4.298	0.911	0.505	0.594	Q1 (sparse)
OCC_Medial	CIN_Anterior	0.027	0.004	4.120	4.682	0.515	0.099	0.584	Q1 (sparse)
OCC_Medial	CIN_Posterior	2.704	0.163	5.244	5.291	0.980	0.554	0.574	Q2
CIN_Posterior	FRO_Prefrontal	0.009	0.471	5.338	11.359	0.366	0.802	0.564	Q3
TEM_Superior	TEM_Medial	0.495	0.042	1.312	10.003	0.782	0.337	0.554	Q1 (sparse)
TEM_Medial	CIN_Posterior	0.057	0.006	1.312	3.325	0.594	0.139	0.545	Q1 (sparse)
FRO_Prefrontal	TEM_Inferior	0.000	0.221	4.359	10.869	0.158	0.624	0.535	Q2
TEM_Inferior	OCC_Lateral	0.000	0.165	4.359	5.821	0.099	0.574	0.525	Q2
TEM_Medial	PAR	0.000	0.194	1.312	15.841	0.139	0.614	0.525	Q1 (sparse)
OCC_Lateral	TEM_Medial	2.211	0.116	1.312	4.298	0.950	0.475	0.525	Q1 (sparse)
CIN_Anterior	OCC_Medial	0.033	0.000	4.120	4.682	0.535	0.050	0.515	Q1 (sparse)
FRO_Prefrontal	PAR	0.001	0.340	17.380	23.874	0.208	0.703	0.505	Q4 (dense)
TEM_Superior	FRO_Prefrontal	0.006	0.574	17.380	18.037	0.337	0.851	0.485	Q4 (dense)
TEM_Inferior	PAR	0.000	0.268	4.359	17.364	0.109	0.644	0.465	Q2
OCC_Medial	TEM_Medial	0.465	0.010	1.312	3.278	0.772	0.208	0.436	Q1 (sparse)
CIN_Posterior	OCC_Lateral	0.297	0.004	5.338	6.311	0.733	0.109	0.376	Q3

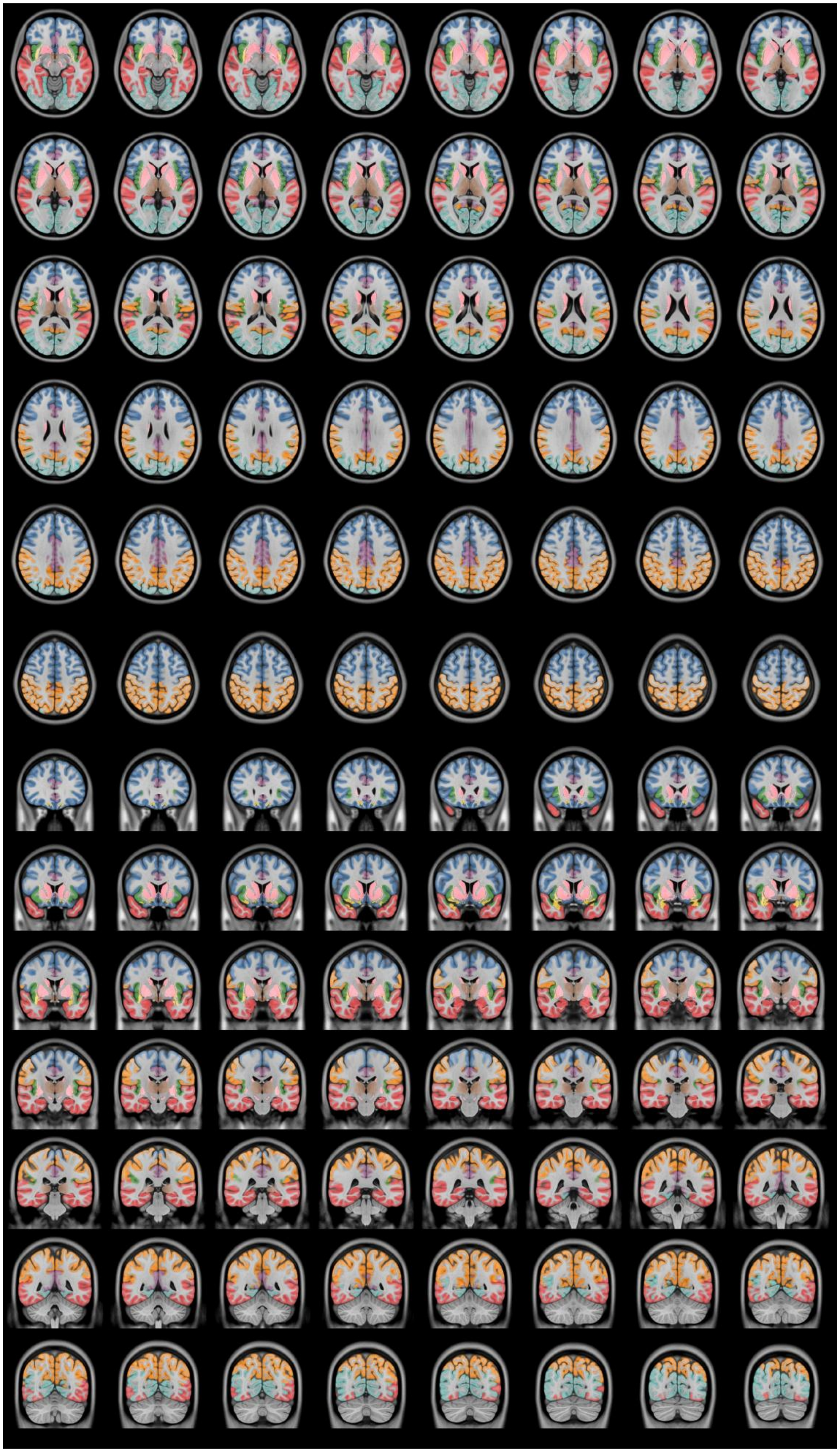

Supplementary Figure 1 | Human atlas mosaic views.
Axial and coronal mosaic views of the Level-1 cortical parcellation in the human brain. Each color corresponds to a macro-region as defined in the common cross-species atlas and shown in Figure 2 (frontal, parietal, temporal, occipital, insular, cingulate, basal ganglia, thalamic, and olfactory divisions). Renderings highlight the spatial extent and anatomical boundaries of these divisions in stereotaxic coordinates.

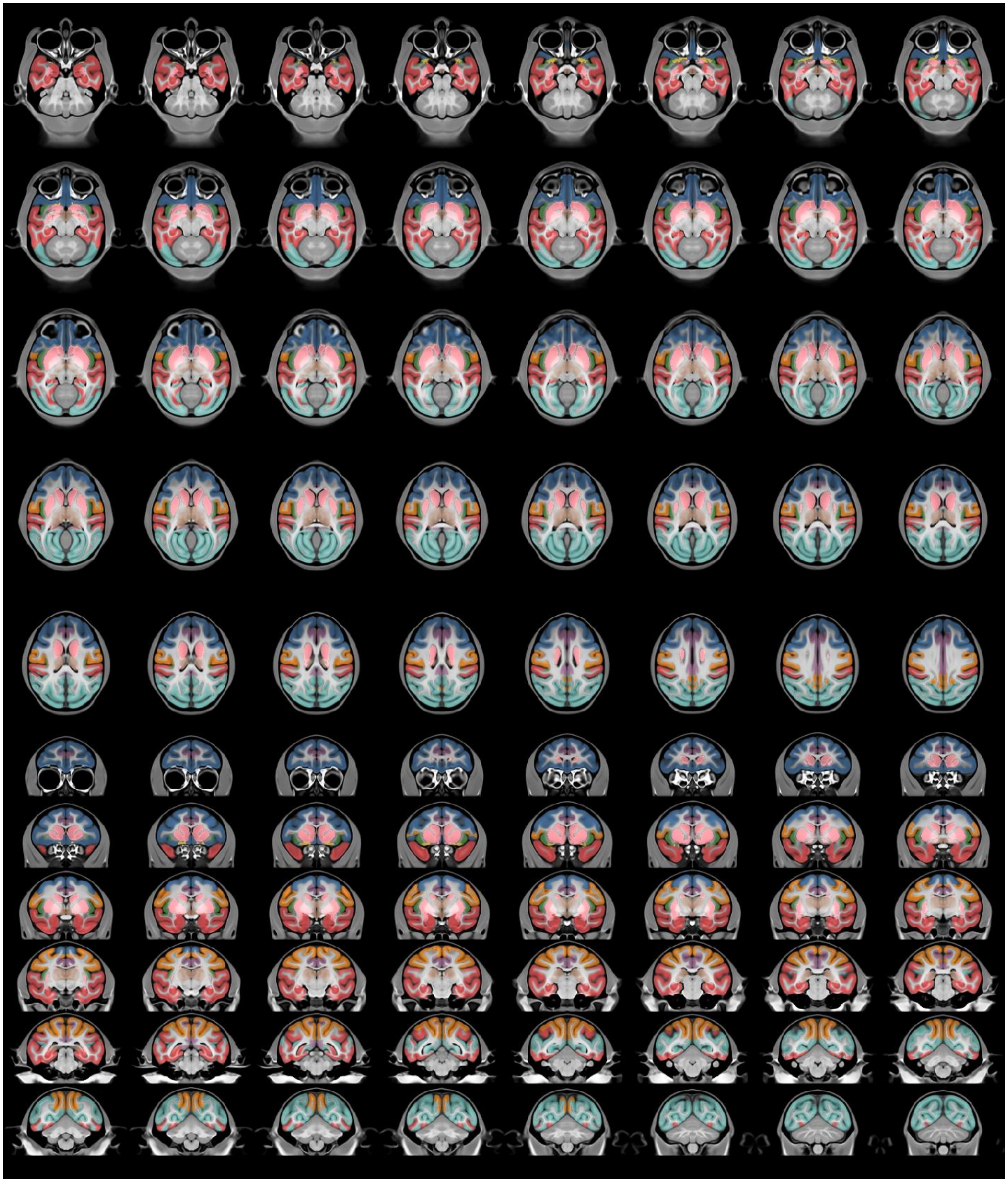

Supplementary Figure 2 | Rhesus macaque atlas mosaic views.
Axial and coronal views of the Level-1 parcellation in rhesus macaque, using the same color scheme as Figure 2. Mosaics illustrate overall alignment of macro-regions with human homologues while showing species-specific sulcal and gyral morphology.

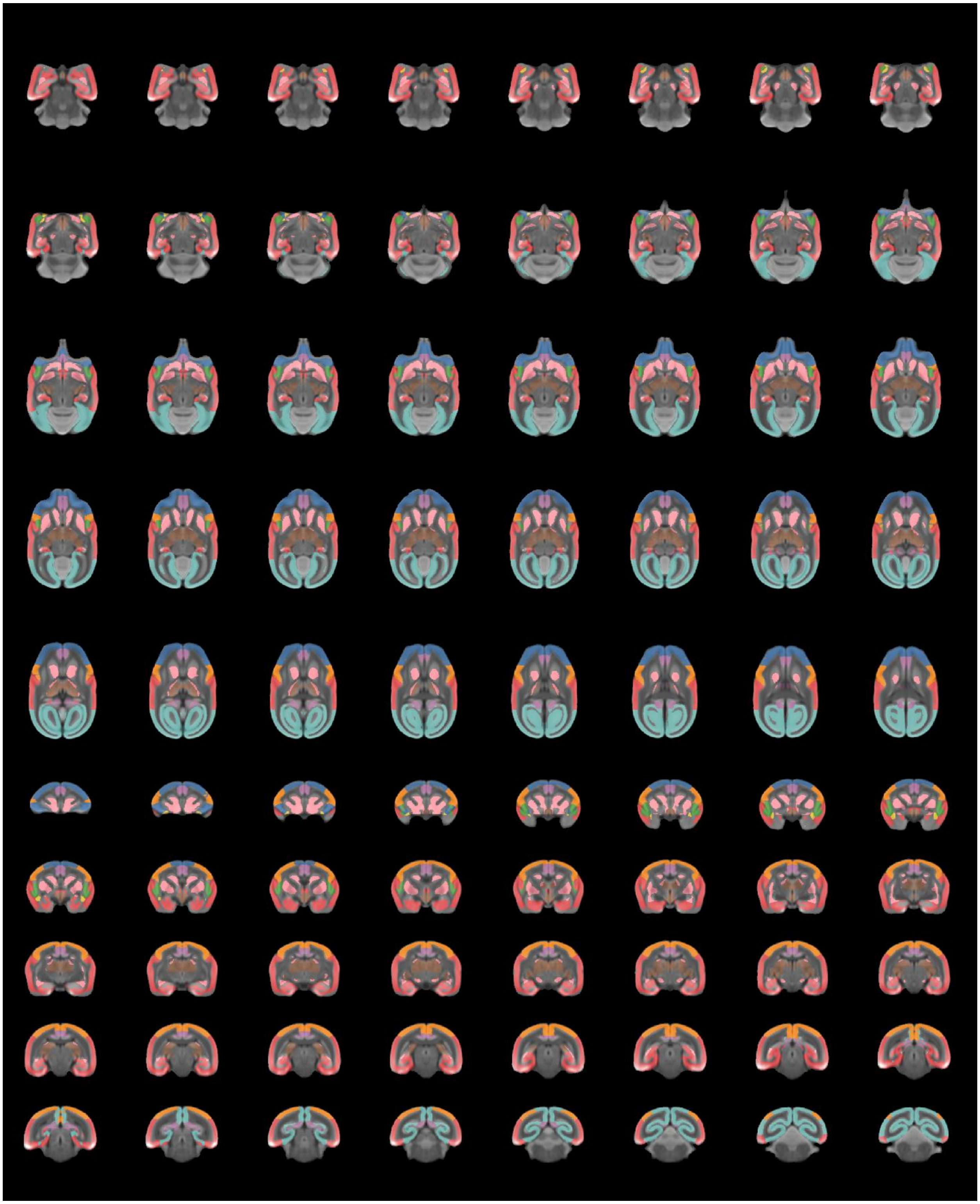

Supplementary Figure 3 | Marmoset atlas mosaic views.
Level-1 parcellation of the marmoset brain displayed in axial and coronal mosaics. Colors follow the common scheme in Figure 2, emphasizing conserved macro-organization and the more compact cortical layout characteristic of New World primates.

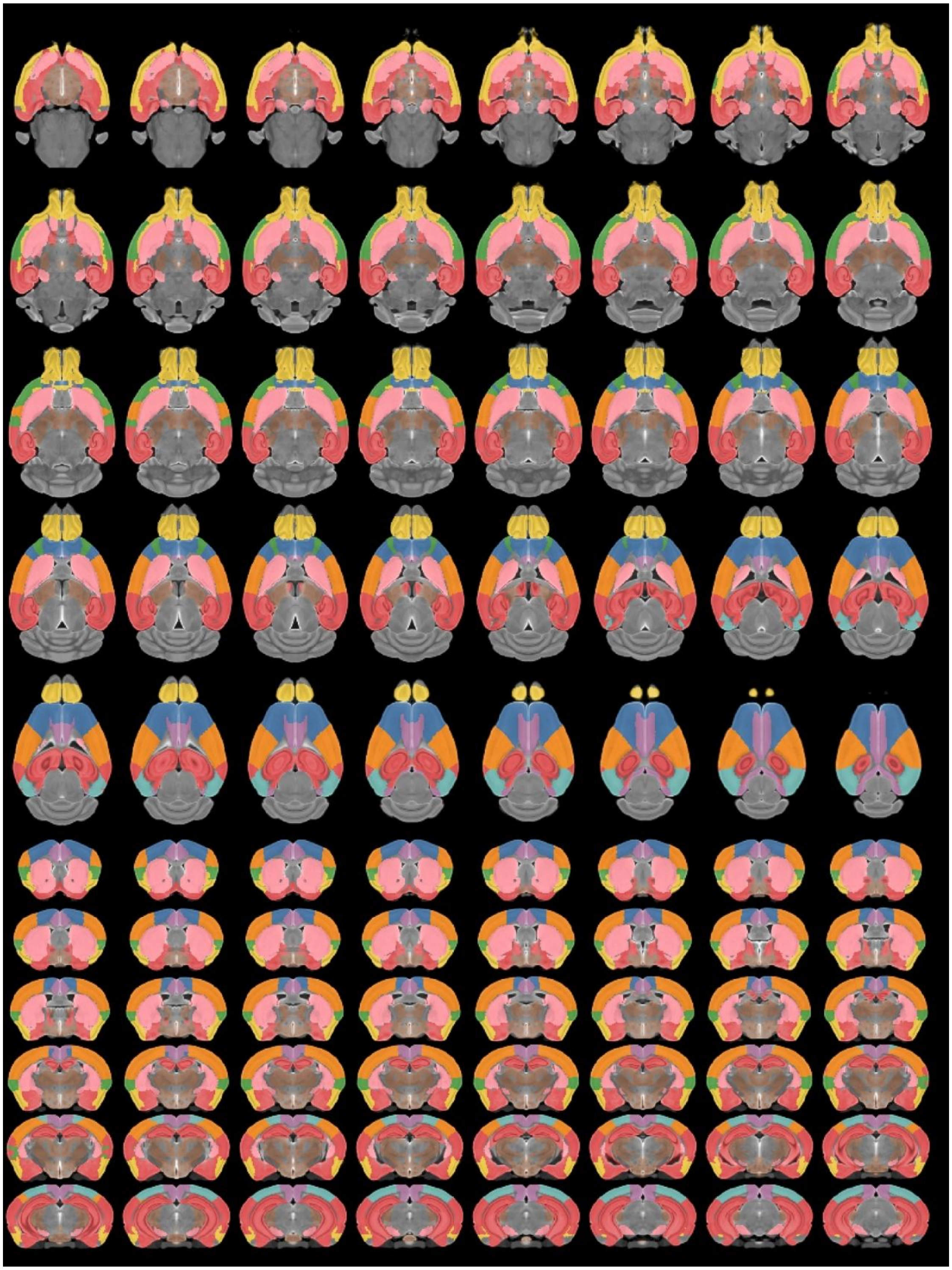

Supplementary Figure 4 | Mouse atlas mosaic views.
Axial and coronal mosaic views of the Level-1 parcellation in the mouse brain. Colors correspond to the same macro-regions used in Figure 2, demonstrating cross-species continuity of frontal, parietal, temporal, occipital, insular, cingulate, basal ganglia, thalamic, and olfactory divisions within the shared atlas framework.

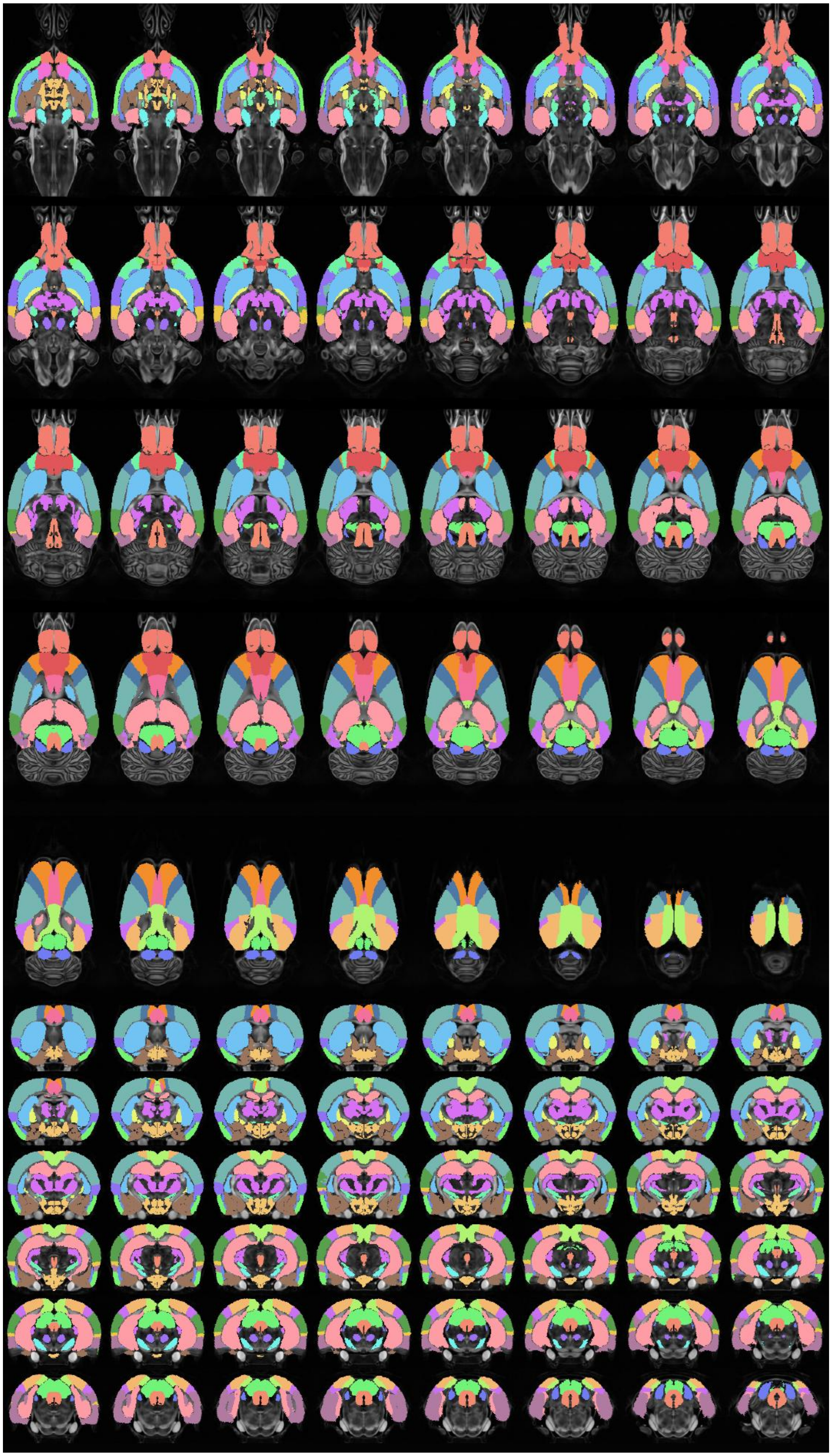

Supplementary Figure 5 | Rat atlas mosaic views. Axial and coronal mosaic views of the Level-1 parcellation in the rat brain. Rat parcellations were derived by warping the mouse CHA using MRI-based nonlinear registration, as described in Methods.

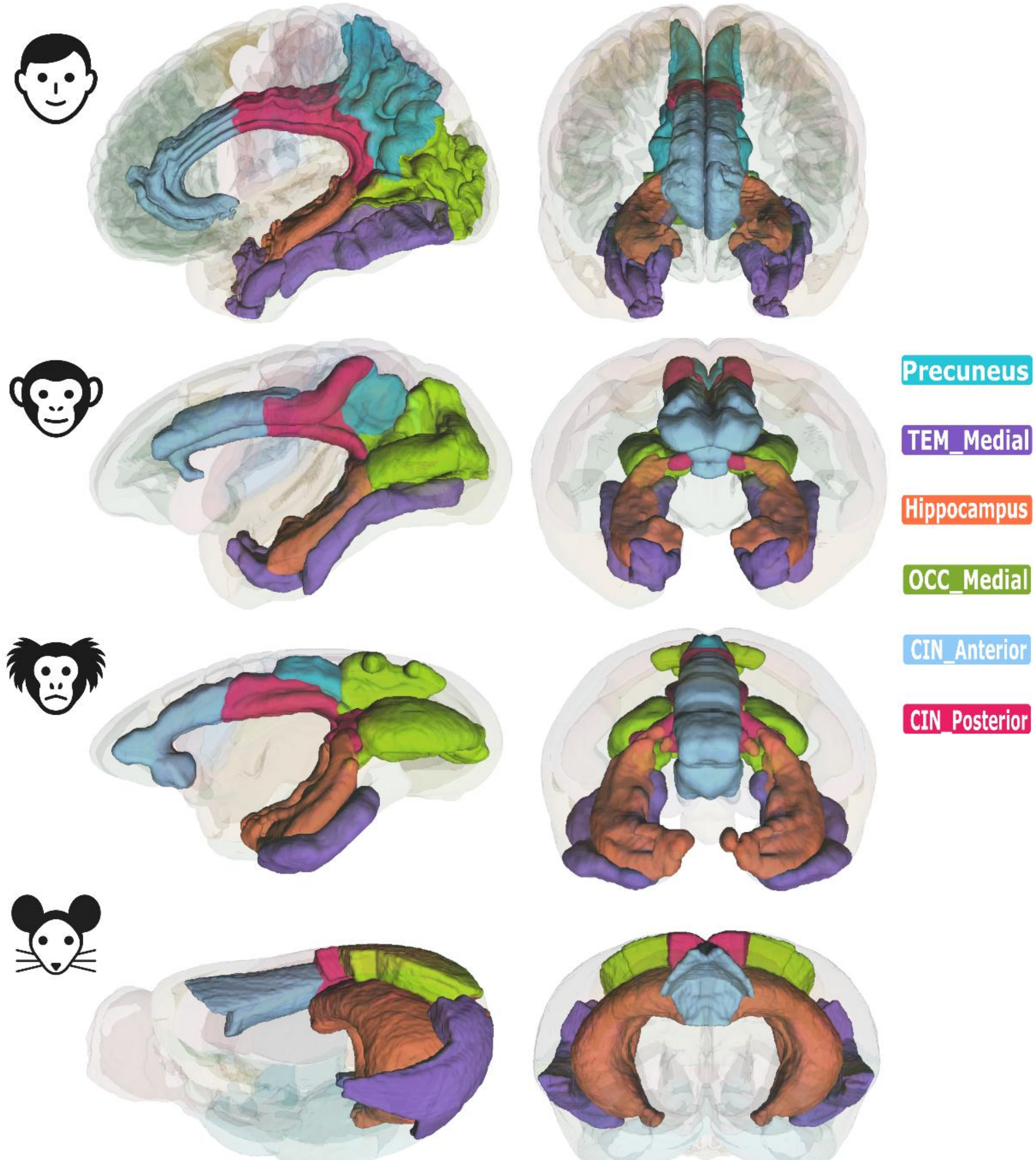

Supplementary Figure 6 | Medial structures across species. Three-dimensional renderings of medial CHA regions shown in sagittal (left) and coronal (right) views for human, rhesus macaque, marmoset, and mouse. Regions displayed: precuneus (cyan; primates only), medial temporal cortex (purple), hippocampus (orange), medial occipital cortex (green), anterior cingulate (light blue), and posterior cingulate (pink). The dorsomedial-to-ventromedial repositioning of posterior cingulate from rodent to primate is apparent in both views, consistent with known cortical folding differences and the low geometric consistency for the CIN_Posterior-PAR pair (R = 0.36; Figure 7a). Brain surfaces are rendered semi-transparently to reveal the depth of medial structures.

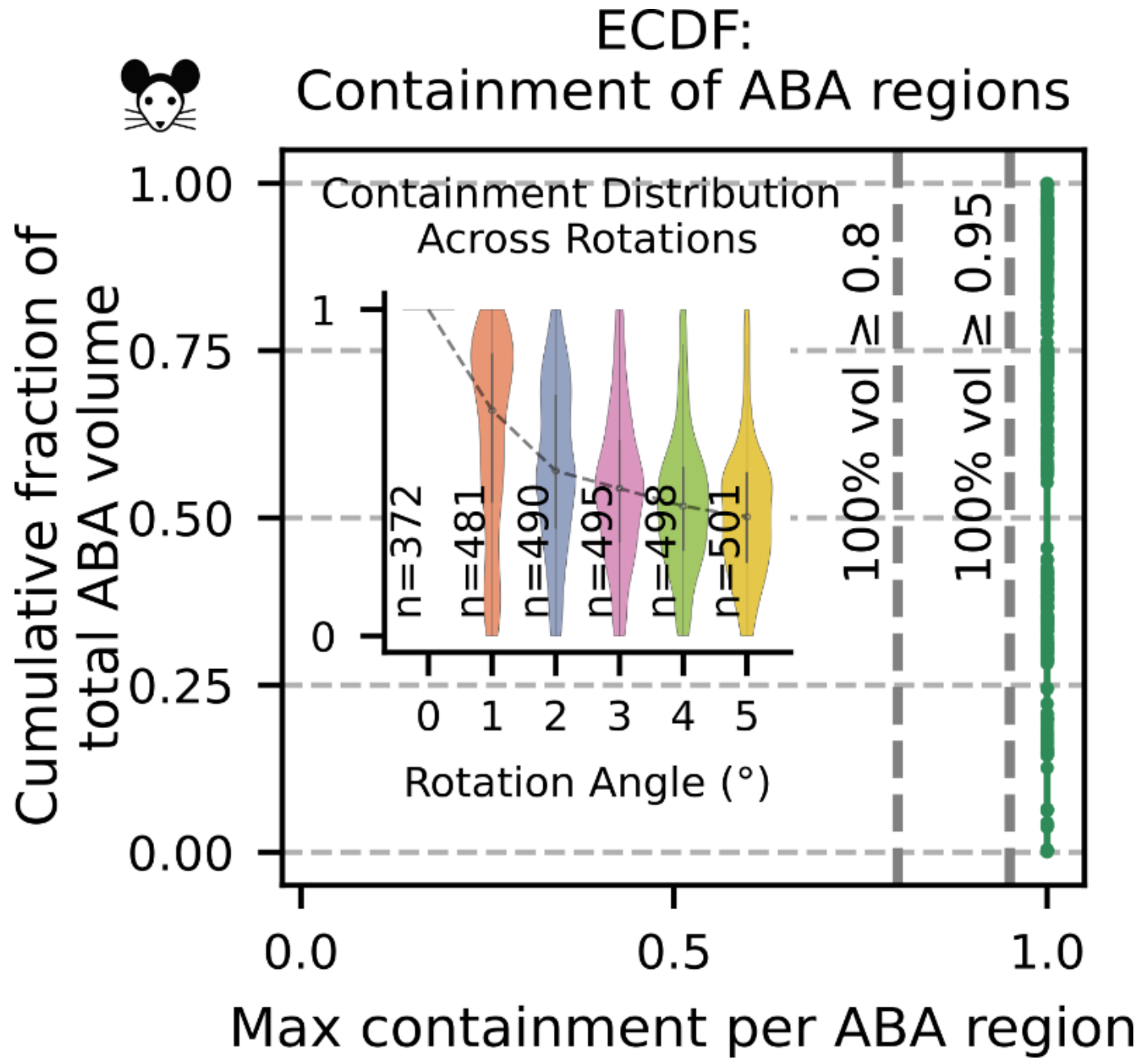

Supplementary Figure 7 | Mouse containment validation. Volume-weighted ECDF of containment scores for Allen Brain Atlas (ABA) regions within CHA. All regions achieved perfect containment (score = 1.0), as expected given that ABA served as the sole source for CHA mouse labels (no boundary loss). Rotation perturbation analysis (inset) demonstrates systematic decay of containment with increasing spatial disruption, validating the sensitivity of the metric. Insets show region-wise containment scores at each rotational-perturbation angle as violin plots (kernel-density estimates; n indicated per violin). Each violin contains an internal box plot: centre line, median; box bounds, 25th and 75th percentiles (interquartile range, IQR); whiskers, 1.5 × IQR.
