# Supplementary material for: A hierarchical framework for cortical and subcortical gray-matter parcellation across rodents, primates, and humans
Source: Nat Commun. 2026 Jul 23;17:8964. doi: 10.1038/s41467-026-75837-5 (PMC13503721; doi:10.1038/s41467-026-75837-5)
Supplement: Supplementary file 2 [file 41467_2026_75837_MOESM2_ESM.pdf]

Description of Additional Supplementary Files

File name: Supplementary Data 1
Description: The Common Hierarchical Atlas: labelled gray-matter parcellations for five species; the constructed minimal deformation templates for mouse, rat and rhesus macaque; and the AC-centered marmoset reference template (MBM v3).
