# Supplementary material for: A hierarchical framework for cortical and subcortical gray-matter parcellation across rodents, primates, and humans
Source: Nat Commun. 2026 Jul 23;17:8964. doi: 10.1038/s41467-026-75837-5 (PMC13503721; doi:10.1038/s41467-026-75837-5)
Supplement: Supplementary file 3 [file 41467_2026_75837_MOESM4_ESM.pdf]

nature portfolio

Corresponding author(s): Siva Venkadesh; Fang-Cheng Yeh

Last updated by author(s): Jun 25, 2026

Reporting Summary

Nature Portfolio wishes to improve the reproducibility of the work that we publish. This form provides structure for consistency and transparency in reporting. For further information on Nature Portfolio policies, see our Editorial Policies and the Editorial Policy Checklist.

Statistics

For all statistical analyses, confirm that the following items are present in the figure legend, table legend, main text, or Methods section.

n/a	Confirmed	
☐	☒	The exact sample size (*n*) for each experimental group/condition, given as a discrete number and unit of measurement
☐	☒	A statement on whether measurements were taken from distinct samples or whether the same sample was measured repeatedly
☐	☒	The statistical test(s) used AND whether they are one- or two-sided<br>*Only common tests should be described solely by name; describe more complex techniques in the Methods section.*
☒	☐	A description of all covariates tested
☐	☒	A description of any assumptions or corrections, such as tests of normality and adjustment for multiple comparisons
☐	☒	A full description of the statistical parameters including central tendency (e.g. means) or other basic estimates (e.g. regression coefficient) AND variation (e.g. standard deviation) or associated estimates of uncertainty (e.g. confidence intervals)
☐	☒	For null hypothesis testing, the test statistic (e.g. *F*, *t*, *r*) with confidence intervals, effect sizes, degrees of freedom and *P* value noted<br>*Give P values as exact values whenever suitable.*
☒	☐	For Bayesian analysis, information on the choice of priors and Markov chain Monte Carlo settings
☐	☒	For hierarchical and complex designs, identification of the appropriate level for tests and full reporting of outcomes
☐	☒	Estimates of effect sizes (e.g. Cohen's *d*, Pearson's *r*), indicating how they were calculated

Our web collection on statistics for biologists contains articles on many of the points above.

Software and code

Policy information about availability of computer code

Data collection	No data were collected for this study; all MRI datasets were previously acquired by others and are described in the Data availability section. No data-collection software was used by the authors.
Data analysis	U-NET Studio v2023.04.17 (https://unet-studio.labsolver.org); DSI Studio v2024.05.22 and later; Python 3.11 with NumPy 1.24.4, SciPy 1.10.1, pandas 1.3.3, Matplotlib 3.7.5, seaborn 0.11.2, and nibabel 3.2.1; ANTs 2022-10-12 commit 71c98905; BART v0.7.00-536-g6be1997; SAMBA CIVM fork, core MDT algorithm identical to the public release. All custom code are publicly available at Zenodo (https://doi.org/10.5281/zenodo.17653308) in the directory projects/common_cross_species_atlas/.

For manuscripts utilizing custom algorithms or software that are central to the research but not yet described in published literature, software must be made available to editors and reviewers. We strongly encourage code deposition in a community repository (e.g. GitHub). See the Nature Portfolio guidelines for submitting code & software for further information.

Data

Policy information about availability of data

All manuscripts must include a data availability statement. This statement should provide the following information, where applicable:

- Accession codes, unique identifiers, or web links for publicly available datasets
- A description of any restrictions on data availability
- For clinical datasets or third party data, please ensure that the statement adheres to our policy

The Common Hierarchical Atlas (CHA) generated in this study is provided as Supplementary Data with this paper.

The source datasets analysed in this study are publicly available: the ICBM 2009a human template (ref 9); the Brain/MINDS Marmoset Brain MRI dataset NA216 (DOI 10.24475/bminds.mri.thj.4624; ref 53) and the Marmoset Brain Mapping v3 templates (ref 10); rhesus macaque images from the PRIMatE Data Exchange (PRIME-DE) via the International Neuroimaging Data-sharing Initiative (ref 54); the Allen Mouse Brain Connectivity Atlas (connectivity.brain-map.org; ref 29); the Marmoset Brain Connectivity Atlas (marmosetbrain.org; ref 30); the Waxholm Space rat atlas (WHS v4.01; refs 27, 45); the Duke Wistar rat atlas (ref 28); the Neuroparc human parcellation repository (ref 4); and the reference atlases used for homology mapping (Allen Brain Atlas, ref 43; CIVM rhesus, ref 60; INIA19-NeuroMaps, ref 61; Brain/MINDS marmoset, ref 62; MBM, ref 63; Paxinos, ref 64; FreeSurfer, refs 57, 58; Brodmann, ref 59).

The mouse and rat diffusion MRI used to construct the templates were acquired at the Duke Center for In Vivo Microscopy and are described in refs 46 and 47; no new animal data were generated for this study.

The source data underlying Figs 4–7 are openly available as .csv and .txt files in the repository deposited at Zenodo (see Code Availability), and a documented Jupyter notebook with figure-labelled cells regenerates all panels from these source files.

Research involving human participants, their data, or biological material

Policy information about studies with human participants or human data. See also policy information about sex, gender (identity/presentation), and sexual orientation and race, ethnicity and racism.

Reporting on sex and gender	The human data are the publicly available ICBM152 population-average template (152 adults, 18–44 y), used solely as a coordinate space; no new human participants were enrolled, and consent and compensation were the responsibility of the original release. As no individual-level human analysis was performed, no sex/gender analysis applies
Reporting on race, ethnicity, or other socially relevant groupings	NA
Population characteristics	NA
Recruitment	NA
Ethics oversight	NA

Note that full information on the approval of the study protocol must also be provided in the manuscript.

Field-specific reporting

Please select the one below that is the best fit for your research. If you are not sure, read the appropriate sections before making your selection.

[x] Life sciences [ ] Behavioural & social sciences [ ] Ecological, evolutionary & environmental sciences

For a reference copy of the document with all sections, see nature.com/documents/nr-reporting-summary-flat.pdf

Life sciences study design

All studies must disclose on these points even when the disclosure is negative.

Sample size	No new experimental data were collected for this study. The atlas was derived from publicly available and previously published high-resolution MRI datasets representing five species (human, rhesus macaque, marmoset, rat, and mouse). Template construction used representative population averages described in the Methods section. Sample sizes reflect those used to generate each species' template: 152 human scans, 35 rhesus macaque scans, 126 marmoset scans, 18 rat brains, and 22 mouse brains. These sample sizes were determined by the availability of high-quality data in each dataset and are consistent with previous cross-species neuroimaging studies. Mouse and rat cohorts were male-only, which is acknowledged as a limitation in the Discussion.
Data exclusions	Dice validation in Figure 5a-b systematically excluded low-performing Neuroparc atlases for each region. The low-performance was defined as having an average dice overlap of less than 0.1 across other Neuroparc atlases. Four cortical regions and subcortical regions were excluded from connectivity validation, as marmoset tracer data lacked coverage in these areas. These are explicitly discussed in the manuscript.
Replication	All analyses were performed using open-source code available in the project's public GitHub repository archived at Zenodo. Dice validation first replicated previously published measures using Neuroparc atlases, which provided a benchmark for the common atlas. Because the study is based on publicly available data and deterministic procedures, replication is ensured by re-execution of the provided code.
Randomization	No randomization was required because this study did not involve experimental groups, interventions, or behavioral comparisons. All datasets were processed using standardized, deterministic preprocessing and atlas-registration pipelines applied uniformly across species.
Blinding	Blinding was not applicable because all analyses were performed on de-identified, publicly available datasets and involved automated computational processing without manual scoring or intervention.

Reporting for specific materials, systems and methods

We require information from authors about some types of materials, experimental systems and methods used in many studies. Here, indicate whether each material, system or method listed is relevant to your study. If you are not sure if a list item applies to your research, read the appropriate section before selecting a response.

Materials & experimental systems

n/a	Involved in the study
☒	☐ Antibodies
☒	☐ Eukaryotic cell lines
☒	☐ Palaeontology and archaeology
☐	☒ Animals and other organisms
☒	☐ Clinical data
☒	☐ Dual use research of concern
☒	☐ Plants

Methods

n/a	Involved in the study
☒	☐ ChIP-seq
☒	☐ Flow cytometry
☐	☒ MRI-based neuroimaging

Animals and other research organisms

Policy information about studies involving animals; ARRIVE guidelines recommended for reporting animal research, and Sex and Gender in Research

Laboratory animals	Mouse, Mus musculus, C57BL/6J, male, n = 22, approximately 90 days at acquisition. Rat, Rattus norvegicus, Wistar, male, n = 18, approximately P90.
Wild animals	Not applicable
Reporting on sex	The mouse cohort (C57BL/6J, n=22) and rat cohort (Wistar, n=18) used for MDT construction were male-only, reflecting the composition of the source acquisitions Sex was not a variable in the study design, as analyses were performed on population-averaged templates rather than individual subjects; however, the use of male-only rodent cohorts may influence volumetric scaling and is acknowledged as a limitation in the Discussion (Section 3.7). The marmoset, rhesus macaque, and human templates were derived from mixed-sex cohorts as provided by their respective source repositories. Sex-disaggregated analyses were not performed because the study operates at the level of species-averaged templates, not individual subjects.
Field-collected samples	Not applicable
Ethics oversight	Duke University IACUC (rodent protocol A169-20-08)

Note that full information on the approval of the study protocol must also be provided in the manuscript.

Plants

Seed stocks	NA
Novel plant genotypes	NA
Authentication	NA

Magnetic resonance imaging

Experimental design

Design type	This is a cross-species neuroimaging study that constructs a hierarchical cortical and subcortical gray matter atlas from population-averaged MRI templates of mouse, rat, marmoset, rhesus macaque, and human brains. The design integrates structural MRI data across species using standardized segmentation and registration methods to ensure anatomical consistency. Validation includes cross-atlas Dice similarity, containment analysis, cross-species invasive tracer connectivity comparison, and a per-region homology confidence index. No new imaging data were acquired for this work.
Design specifications	Atlas generation followed a common hierarchical framework: segmentation into gray matter, white matter, deep nuclei, and cerebrospinal fluid; delineation of nine Level-1 macro-regions based on major anatomical landmarks; and further subdivision into finer Level-2 regions. All species were aligned to their respective stereotaxic coordinate systems using affine and nonlinear registration, ensuring topological consistency across species. Analyses were performed at multiple spatial scales to confirm reproducibility of hierarchical relationships.
Behavioral performance measures	No behavioral data were collected or analyzed in this study. The work focuses on anatomical parcellation and cross-species comparison of cortical organization.

Acquisition

Imaging type(s)	Diffusion MRI. Mouse and rat species templates were constructed from actively-stained, high-resolution diffusion MRI acquired ex vivo. The marmoset, rhesus macaque, and human inputs were existing publicly available templates or datasets (Brain/MINDS MBM v3, PRIMatE Data Exchange, and ICBM152 2009a, respectively) rather than data acquired for this study.
Field strength	9.4 T for mouse, 7 T for rat for the diffusion acquisitions. Field strengths for marmoset, rhesus macaque, and human reflect the acquisition specifications of the source datasets.
Sequence & imaging parameters	Mouse: Stejskal–Tanner spin-echo diffusion sequence with compressed sensing, 25 µm isotropic resolution; 61 diffusion-weighted volumes at b = 3000 s/mm² and 6 baselines (TR = 80 ms, TE = 14 ms), uniformly distributed gradient directions, on a 9.4 T vertical Oxford magnet. Rat: Stejskal–Tanner spin-echo diffusion sequence, b = 3000 s/mm² with 61 directions and periodic baselines, on a Bruker 7 T Avance III. Both acquisitions used compressed-sensing acceleration and were reconstructed with generalized q-sampling imaging (GQI) in DSI Studio. The marmoset, rhesus macaque, and human inputs were existing public templates or datasets at their original acquisition specifications and were not acquired for this study.
Area of acquisition	Whole brain

Diffusion MRI ☒ Used ☐ Not used

Parameters: single-shell, b = 3000 s/mm², 61 directions, plus 6 baselines, reconstructed with GQI in DSI Studio.

Preprocessing

Preprocessing software	ANTs 2022-10-12 commit 71c98905; BART v0.7.00-536-g6be1997; SAMBA CIVM fork 2020, core MDT algorithm identical to the public release.
Normalization	Population-averaged minimal deformation templates were constructed per species via iterative diffeomorphic averaging (ANTs; SAMBA for rat). Tissue compartments were segmented with U-NET Studio, and the hierarchical parcellation was defined in DSI Studio. No subject-level normalization was performed, as the study operates on population templates.
Normalization template	The human template was adopted from 152 healthy adult MRI scans; the rhesus macaque template was generated from 35 scans; the marmoset template was adopted from 126 scans; the rat template was generated from 18 Wistar brains; and the mouse template was generated from 22 C57BL/6 brains. All templates were aligned to standard stereotaxic coordinate systems with the origin at the anterior commissure and AC-PC alignment. The normalization procedures and template construction are described in detail in the manuscript Methods.
Noise and artifact removal	Standard preprocessing of the source datasets included eddy-current correction, motion correction, and gradient nonlinearity correction. No additional artifact removal beyond these published procedures was performed. These procedures are described in detail in manuscript methods.
Volume censoring	Not applicable; all volumes provided by the source datasets passed their original quality-control filters.

Statistical modeling & inference

Model type and settings	Quantitative validation employed both descriptive spatial metrics and inferential statistics. Cross-atlas Dice similarity was tested using a one-sided Wilcoxon signed-rank test across regions (median delta = +0.125, 95% CI [0.070, 0.206], p = 1.22 x 10^-4). Cross-species connectivity correspondence was assessed using Spearman rank correlation between mouse anterograde and marmoset retrograde tracer matrices mapped to CHA (rho = 0.61, p = 2.05 x 10^-11). Containment validation used a volume-weighted, chance-adjusted containment metric analogous to the adjusted Rand index, with perturbation analysis confirming sensitivity to spatial disruption. Per-region homology confidence was computed as a composite index integrating nomenclature directness, geometric consistency, and connectivity correspondence. All analyses were implemented in Python using deterministic, reproducible procedures available in the public code repository.
Effect(s) tested	The Wilcoxon signed-rank test assessed whether CHA achieves spatial overlap with established human atlases at least equivalent to inter-atlas agreement. The Spearman correlation tested rank-order correspondence of directed connection weights between mouse and marmoset tracer datasets mapped to CHA. Containment validation quantified boundary alignment between CHA and finer-scale species-specific atlases, with a rat inter-atlas baseline contextualizing results. Perturbation analysis confirmed that containment scores degrade under spatial disruption, validating the metric's sensitivity.

Specify type of analysis: ☐ Whole brain ☐ ROI-based ☒ Both

Anatomical location(s): For each Level-2 region, homologues were identified from one or more standard atlases: FreeSurferSeg (human), Brodmann (human), the Allen Brain Atlas (mouse), CIVM rhesus atlas (rhesus macaque), Inia19–NeuroMaps (rhesus macaque), Brain/MINDS (marmoset), MBM atlas (marmoset), and Paxinos (marmoset) as explained in Methods section in detail.

Statistic type for inference (See Eklund et al. 2016)	Inferential statistics were used for two validation analyses: a one-sided Wilcoxon signed-rank test to assess whether CHA Dice overlap with established human atlases meets or exceeds inter-atlas agreement, and a Spearman rank correlation to test cross-species correspondence between mouse and marmoset tracer connectivity matrices mapped to CHA. Containment validation and the homology confidence index are descriptive metrics that do not involve hypothesis testing.
Correction	Not applicable

Models & analysis

n/a	Involved in the study
☒	☐ Functional and/or effective connectivity
☒	☐ Graph analysis
☒	☐ Multivariate modeling or predictive analysis
