# Supplementary material for: A hierarchical framework for cortical and subcortical gray-matter parcellation across rodents, primates, and humans
Source: Nat Commun. 2026 Jul 23;17:8964. doi: 10.1038/s41467-026-75837-5 (PMC13503721; doi:10.1038/s41467-026-75837-5)
Supplement: Supplementary file 4 [file 41467_2026_75837_MOESM5_ESM.pdf]

nature portfolio

Peer Review File

A hierarchical framework for cortical and subcortical gray-matter parcellation across rodents, primates, and humans

Corresponding Author: Dr Siva Venkadesh

This file contains all reviewer reports in order by version, followed by all author rebuttals in order by version.

Version 0:

Reviewer comments:

Reviewer #1

(Remarks to the Author)
The authors present an ambitious study which aims to simplify translation from four preclinical models through a common atlas with human-centric nomenclature. To this end the authors create a hierarchical atlas, starting from simple tissue segmentation maps and then move towards more general lobes and then fine-grained parcellations with each species. While the author's work appears to be methodologically sound, there are significant limitations in the presentation and interpretation of their results.

Major Points
1. These initial subdivisions were defined as "Subdivisions were primarily established in humans using high-resolution MRI and aligned with anatomical conventions from widely used atlases." Given the importance of other atlases in defining level-1 parcels Table 1 should have greater prominence. Right now, it is only shown in the methods and not present in the main text. The human subdivisions are not controversial, however the way this initial sub-division is defined inherently sets the stage for all subsequent analysis and naming given the "human-centric" approach. To understand the reliability of this, a clear set of criteria used to define the level-1 parcels and a more in-depth description should be provided for each species.

2. The authors attempt to validate their findings based on containment criteria of similarly named level-2 areas in the level-1 atlas. This is inherently circular and qualitative as they are validating different levels of the hierarchy based on whether they belong to one another across species. Yet level-1 subdivisions are not externally validated aside from atlas criteria which as mentioned in my first comment must be clearly specified for each species. Effectively, in each species they are using different levels of their own outputs to validate their outputs from another level of the atlas. I would propose an external validation criterion for the subdivisions made, not based solely on volumetry. This has recently been done quite extensively in the comparative neuroanatomy literature through connectivity (Xu et. al, 2020, Mars et. al, 2018), structure (Eichert et al, 2020) and transcriptomic features (Beauchamp et al. 2022).

These approaches enable quantitative measures of how similar areas are across species based on features of the brain itself, and would provide sufficient validation to better ascertain the level of homogeneity claimed in each sub-division at either level-1 or level-

While this may be impractical to do for all of the species used in the paper, cross species features and alignments are available for macaques (Xu et al., 2020, Mars et al. 2018, Warrington et al. 2020), and for mice (Beauchamp et al. 2022). I would ask the authors to use these resources to provide quantitative, non-volumetry validation of the proposed homologies.

In short, the authors claim cross-species alignment of parcels based on a standardized anatomical framework. Yet the framework itself eschews actual quantitative alignment, and instead relies on the definitions of regions defined by prior atlases, and whether or not they belong to the intermediate parcel class defined by the authors through a circular containment criteria.

3. While aiming for a common atlas is an admirable goal, it would be nice for the authors to discuss the appropriateness of the approach in comparative neuroanatomy. A specific and concrete example of this is presented in the discussion, "the mouse PAR parcel corresponds primarily to its continuous somatosensory territory, whereas primates possess a subdivided parietal cortex comprising postcentral, superior, inferior, and precuneus regions."

I would ask the authors to extend and further justify their choice to call this the parietal cortex in the mouse. While I understand the aim is to provide a human-centric naming convention, calling somatosensory cortex parietal cortex is inherently misleading. In humans the parietal cortex contains major association hubs where sensory integration and multimodal processing occur (Wei et al. 2024). Calling somatosensory cortex the parietal cortex in this case is misleading both structurally and functionally.

I do not understand how such a mismatch would not lead to further confusion in cross-species nomenclature and translation. If a mouse derived result is localized to the somatosensory cortex, then why would I report it as in the parietal cortex defined by the authors? Doing so now implies that my results occur in an area where multisensory integration occurs; extending associative function to a part of the cortex based solely on nomenclature. This is the intrinsic problem with renaming animal regions in a human-centric fashion without validation across multiple methods. Overall, when performing cross-species analysis it may be more precise to leave an imprecise match out rather then enforcing a nomenclature which does not fit the region it is assigned as it will only cause more confusion. The authors do respect this in the Acumbens, so why not for other regions where no clear match may exist?

4. The authors claim, “whereas rhesus macaques exhibited a relatively greater investment in temporal regions.” Relative to which species? It has been previously shown that humans do have greater temporal innervation and expansion relative to other primates (Eichert et al. 2020). I would implore the authors to please write with the maximum amount for clarity which species is being compared to which in sentences such as this.

5. The rat was treated as a non-core species? Why? Multiple rat atlases including the Waxholm rat atlas exist and are freely available? https://ebrains.eu/data-tools-services/brain-atlases/rat-brain. It does not seem fair to simply assume that a mouse to rat registration should suffice when claiming homology across five different species, and an atlas for that species does exist.

6. The author’s claim that a significant contribution of their work is the construction of templates used in their study. While building templates for their specific study population of rodents and macaques is likely helpful to their site-specific scanner, there are well established and widely used volumetric macaque/marmoset templates (https://prime-re.github.io/templates_and_atlases), and mouse templates (https://imaging.org.au/AMBMC/ https://www.science.org/doi/10.1126/sciadv.adq8089). Furthermore, upon looking at the details of the acquisition, number of animals and the source of the mice, is the mouse atlas the Duke mouse brain atlas? It appears to be the same IACUCU approval, and the same data. In sum, given that a very similar mouse MDT exists, and the general availability of MDTs available the emphasis on the authors MDTs as novel findings are generally overstated.

Minor points
1. Visualization of some figures is confusing and incomplete to provide full context for the regions being shown. This is most evident in figure 3. Specifically, we are only shown axial views. This precludes the ability to assess the depth of medial structures such as the precuneus and cingulate. These views are key to actually understanding their position in the cortex.
2. The U-net results look fantastic and after going to the cited paper I am likely to use this tool myself. However, in the manuscript it would serve to explicitly state that it has been previously trained and validated in these species.
3. The definition of containment score come far too late in the paper. It would serve the readability of the paper to explain it clearly in the main text instead of just the online methods.
4. Similarly, the rotation results are shown without any methodological contextualization. While I understand the ordering format of the paper, it would be good for this analysis to be partially explained while presenting the results.

Final comments:
The authors have taken an ambitious approach to provide a tool to harmonize atlas nomenclature for cross-species translation. While methodologically sound, further contextualization, and precision is needed to describe their findings. This includes exercising greater caution to prevent misnomers harmful to translation that create more confusion than they solve. To this end I would ask for full criteria for each species brought into the main-text, not relegated to the methods. I would also ask for validation of these parcellations in at least macaques and mice based on more than volumetric composition. I would also ask the authors to take into account the resources already available to the comparative neuroanatomy community, and specifically to expound on how their hierarchical atlas solves a problem not settled by using a precise species-specific atlas aligned through quantitative methods across-species?

(Remarks on code availability)
The code ran fine and the notebook for figures was well organized.

Reviewer #2

(Remarks to the Author)
This paper introduces a number of brain templates/atlases based on minimal deformation approaches for different species in an attempt to create a common space for cross-species analysis of developmental motifs, and other comparative approaches. It then uses these atlases to compare relative volumetric data across for some for some coarse brain areas. All data is provided to the scientific in a free and open format.

Cross-species comparison is an important approach for (developmental) neuroscience, and the creation of a common brain

species could facilitate such approach. However, the work presented here, while solid in its underlying methodology, does not completely convince that this is the way to go. This could partially be a matter of presentation and argumentation, but the example brain areas that are compared across species do not seem to yield any new insights, which reduces the potential value of the 'framework' the authors introduce. The core idea of a common hierarchical framework is valid and interesting, but the current paper could use some work to reach its full potential and make it more appealing for the broad readership of Nature Communications.

The cross-species comparison heavily relies on the parcellations of the different species' brains and the assumption of homology. The former is optimized through the MDT approach and a U-Net segmentation for a first coarse segmentation. More refined (yet still relatively coarse) segmentation was then performed based on existing atlases and previously published work on anatomical homologies. In this step, parcels are kept constant as much as possible across species to facilitate comparison. This means that for cross-species comparisons, the parcellations reproduce existing parcels and known homologies which seems to make the discovery of new homologies unlikely. In the validation of the new 'Common Hierarchical Atlas' (CHA), the authors do show that their atlas is more similar to existing atlases than the existing atlases are to each other, meaning it summarizes existing knowledge well. Little or no metrics are provided about the U-Net based segmentation. In the methods section, the authors do refer to an entire preprint on this method by the senior author of this paper that provides such information, but some of this should be included in the current paper.

The creation of the MDTs for each species and the subsequent segmentation seem fine. In the second objective, demonstrating how this information can be used for cross-species comparisons, the paper remains somewhat underwhelming. The authors investigate volumetric composition across species. Parcel volumes are normalized against brain size and relative contributions of parcels to total brain size are compared across species. While this is fine as a first approach, it does not really present a thorough cross-species analysis. What about looking at relative volumes within hierarchical parcels, or at combinations of volumes to investigate brain wide trajectories/patterns in a multivariate way? Would the increased contribution of one area nor automatically lead to the decreased of other areas? This part of the paper should be expanded to yield more meaningful insights.

The result of relatively large contribution of frontoparietal regions in primates and the olfactory system in rodents is not unexpected. Is there any insight the new common hierarchical framework can give us that wasn't already possible with previous atlases?

What is the implication of the validation of the common atlas showing higher cross-atlas dice similarity with other atlases than the other atlases among themselves? Since the CHA parcellation was based on information from all these atlases it makes intuitive sense that it would be more similar to them than atlases that were not based on each other's information. Maybe I'm missing something, but this should be clarified. What is the implication of Fig 5a right panel? It suggests something Weber-Fechner law-like in the sense that regions with low dice coefficients to begin with have smaller differences between CHA and other atlas similarities. Are there outliers regions from the general trend? What does that mean?

The Discussion section summarizes the paper but does not provide a thorough discussion of the potential, limitation, and implications of the new atlas. While some are mentioned, they are mostly repetitions of what was said earlier in the paper. I think that if the value of the framework can be highlighted by additional cross-species analysis that yield new insight, the discussion can be expanded accordingly.

In its current form, the common hierarchical atlas framework presents an interesting approach, with solid underlying methodology for which the demonstration of added value and potential would require some additional work. I believe that if the authors can revise the paper in such a way, it would be a valuable resource for the field of comparative neuroimaging. In its current form it leaves such demonstration to others which strongly reduces the work's impact.

(Remarks on code availability)
I have looked at the code, but I have not reviewed it line-by-line. The code organization looks solid, with ipython notebooks to regenerate the manuscript figures, python code to generate the analyses, and proper documentation. I can only commend the authors for making this available in such an organized format.

Reviewer #3

(Remarks to the Author)
This manuscript presents a novel hierarchical atlas for cortical and subcortical gray-matter regions across rodents (mouse, rat), nonhuman primates (marmoset, rhesus macaque), and humans. The authors construct population-averaged minimal deformation templates (MDTs) for each species to minimize anatomical variability and then apply a 3D U-Net for tissue segmentation (Level 0: GM, WM, DGM, CBL, CSF). They define Level 1 (nine macro-regions, e.g., frontal, parietal) and Level 2 (21–26 subdivisions) using landmark-guided boundaries and literature-based homologies. Validation includes Dice similarity against species-specific atlases and cross-scale containment scores. Volumetric analyses reveal species-specific scaling, such as expanded associative cortices in humans and limbic emphasis in rodents. The atlas is freely available and aims to support comparative neuroscience, connectomics, and translational studies.
This work represents an important contribution to comparative neuroanatomy and addresses a key gap in cross-species brain mapping by integrating data from multiple species into a unified framework. However, there are several unclear points and methodological limitations that require major revision before the manuscript can be considered for acceptance.
While the framework is useful, it lacks clear methodological novelty beyond the integration of existing tools (e.g., ANTs for

MDT construction and U-Net–based segmentation). As noted in the Introduction, several initiatives have already harmonized atlases within primates; extending this approach to rodents is valuable, but the contribution would be more compelling if it incorporated novel alignment or parcellation strategies.
Level-2 parcellation includes only 21–26 regions, which is relatively coarse compared with existing fine-grained atlases (e.g., the Allen Brain Atlas with 372 regions). Although exclusions such as the claustrum, substantia nigra, and subthalamic nucleus are justified by MRI limitations, they reduce whole-brain coverage. In addition, the parietal cortex and caudoputamen remain undivided in rodents, which may oversimplify species-specific organization—particularly in smaller brains, where finer anatomical distinctions are critical. Notably, mouse and rat cohorts are male-only, which may bias volumetric scaling analyses given known sex differences in brain structure.
Although the atlas is literature-guided, some cross-species mappings (e.g., mouse ectorhinal cortex as a homologue of inferior temporal cortex) rely primarily on functional analogies rather than strict anatomical homology, introducing potential subjectivity. Providing confidence scores for each homology assignment or explicitly discussing uncertainty would improve transparency.
Human validation relies primarily on Neuroparc atlases; comparisons with functional parcellations (e.g., Schaefer atlas) would strengthen the claims. Containment scores are substantially lower in marmosets (49% ≥ 0.95), which is attributed to atlas resolution, but this discrepancy warrants deeper analysis of atlas inconsistencies. For nonhuman primates, additional validation using functional connectivity benchmarks or comparisons with other cross-species atlases (e.g., the Chimpanzee Brainnetome) would be valuable. More generally, homology confidence should be quantified on a per-region basis.
No statistical tests are reported to support claims of similarity or superiority (e.g., in Figure 5, where the common atlas is described as providing superior or equivalent overlap). In mouse data, some regions show high similarity, but without statistical testing it is unclear whether this exceeds expectations based on literature-guided segmentation alone. Likewise, volumetric comparisons (e.g., “marmosets exhibited comparatively higher fractions…”) are based on single MDT values per species; mapping regions to individual subject spaces and performing statistical analyses (e.g., ANOVA or permutation tests) would enable more robust inter-species comparisons.
Detailed Questions and Requests for Clarification
• Rat data inconsistencies: The Introduction mentions mouse and rat data, but Figures 1 and 2 only show mouse results. Please clarify this inconsistency. How was the accuracy of rat atlas warping validated? Quantitative metrics (e.g., Dice similarity after warping) should be reported.
• 3D U-Net details: Please clarify the source and training procedure of the 3D U-Net model. If the network was trained on species-specific atlases (as implied by reference 39), this may bias segmentation toward those atlases and compromise independent validation. How was Level-0 segmentation accuracy assessed (e.g., Dice against manual labels)?
• Level 1 and Level 2 segmentation: More detail is needed on how gray matter was subdivided into Level-1 regions (e.g., criteria for separating prefrontal from parietal cortex). For Level-2 regions, clarify whether delineation was manual, automated, or hybrid. While Table 1 is informative, it lacks quantitative criteria for boundary definition.
• Figure 4 (Volumetric composition): Please define “volume fraction” explicitly and describe how it was calculated. Normalization to total brain volume per species is appropriate, but without individual-level data or statistical testing, comparisons based on single values are underpowered.
• Figure 5 clarifications: Please define what constitutes “common” versus “non-common” atlases. No statistical tests are presented to support claims of superior or equivalent performance. How were species-specific atlases aligned to the common atlas for validation, and how was spatial misalignment quantified?
• Inter-rater reliability: For any manual boundary adjustments, inter-rater reliability should be assessed and reported. Providing volumetric values in tabular form would also improve reproducibility and reuse.
• Figures: Improve figure clarity by expanding abbreviations in legends and providing quantitative color-scale thresholds (e.g., in Figure 4b).
• Discussion: The Discussion would benefit from a broader perspective on potential applications, such as use in AI-based segmentation pipelines or integration with transcriptomic (e.g., scRNA-seq) cell-type mapping efforts.

(Remarks on code availability)

Version 1:

Reviewer comments:

Reviewer #1

(Remarks to the Author)
The authors have significantly improved the clarity, readability, and scope of their manuscript through the revisions. I appreciate the move of Table 1 to the main text, even if doing so breaks the flow of the manuscript. By placing the primary findings (and their limitations) up front it is much clearer what they’re CHA actually gives us as a community. Their clarification of the containment criteria and the rewrite of that section have substantially improved my understanding of what was done, and help limit the overclaiming I felt was present previously. I also especially appreciate the care taken to clarify areas that don't match: the paragraphs and parentheticals addressed to mouse researchers are doing real work in providing a clearer manuscript that avoids false equivalency when using this framework.

Overall, the authors have done well to address my prior concerns. On reading this draft, however, I do have one new major point. Could the authors describe how other researchers would integrate a new species, or a new atlas, into the existing framework? I think a common hierarchical atlas can be a useful tool for the community, but only if the broader non-human

neuroimaging community can contribute alternate atlases for the species already included or add new species entirely. Can this be a tool that I can integrate my own data into? Could the authors discuss this, or — better still — implement it? The ability (or at least concrete plans) to extend this framework is, in my view, what determines the long-term impact of work of this kind.

Finally, a few minor points remain:

1. In Table 1, CHA is not defined. I would recommend spelling it out either in the abbreviations list or at first use.

2. The following line in the introduction is unclear: "Furthermore, most existing solutions are based on species-specific surfaces or templates and do not utilize population-averaged minimal deformation templates (MDTs), which are reference spaces created by iteratively averaging multiple individuals to minimize...
MDTs are not species-specific. I suspect the authors mean that most existing resources are based on a single subject, as opposed to a population-level construct like an MDT. The authors seem to clarify this in the next section, but mix terminology of templates and atlases (see next comment). In general, I think too much time is spent on the importance of MDTs. I'd prefer it be a more minor point since it's novelty is limited and they expand on the more novel aspects of their paper.

3. In the line: "In contrast, atlases for other species often rely on single-subject scans or templates not generated using comparable MDT frameworks, resulting in inconsistencies in spatial alignment and anatomical scaling," there is a meaningful distinction between templates and atlases that is being elided. Please be consistent and specific throughout the text about which is which — atlases label regions within a template space. Because this sentence directly follows the description of the human ICBM templates, it is unclear which object (atlas or template) is actually being referred to here.

(Remarks on code availability)

Reviewer #2

(Remarks to the Author)
The authors have thoroughly improved the manuscript with this revision and for me addressed all comments and concerns. The addition of connectivity data and more thorough discussion have made this a much stronger paper.

I only have one comment/question:

In the new Fig 7b, it's a bit confusing how the unavailability of connectivity scores affects the 'homology confidence'. If I understand the methods section correctly, the total score is now basically a division by 2 instead of 3? I'm not convinced this is the best way of accounting for non-available data. Would it not make more sense to score this as a neutral 0.5 and still divide by 3? Are the grey bars in Fig 7B now simply filling up to the average of nomenclature and geometric scores?

(Remarks on code availability)

Reviewer #3

(Remarks to the Author)
The authors have thoroughly addressed all of the reviewer comments in the revised manuscript. The revisions are clear, comprehensive, and significantly improve the quality and presentation of the work. The responses provided are satisfactory, and the changes made demonstrate careful consideration of the feedback. I appreciate the effort invested in strengthening the manuscript. In its current form, I believe the manuscript is suitable for publication.

(Remarks on code availability)

Open Access This Peer Review File is licensed under a Creative Commons Attribution 4.0 International License, which permits use, sharing, adaptation, distribution and reproduction in any medium or format, as long as you give appropriate credit to the original author(s) and the source, provide a link to the Creative Commons license, and indicate if changes were made.
In cases where reviewers are anonymous, credit should be given to 'Anonymous Referee' and the source.
The images or other third party material in this Peer Review File are included in the article's Creative Commons license, unless indicated otherwise in a credit line to the material. If material is not included in the article's Creative Commons license and your intended use is not permitted by statutory regulation or exceeds the permitted use, you will need to obtain permission directly from the copyright holder.
To view a copy of this license, visit https://creativecommons.org/licenses/by/4.0/

Response to Reviewer #1

> *The authors present an ambitious study which aims to simplify translation from four preclinical models through a common atlas with human-centric nomenclature. To this end the authors create a hierarchical atlas, starting from simple tissue segmentation maps and then move towards more general lobes and then fine-grained parcellations with each species. While the author's work appears to be methodologically sound, there are significant limitations in the presentation and interpretation of their results.*

We thank the reviewer for the thorough evaluation and for recognizing the methodological soundness of the work. We have addressed all concerns regarding presentation and interpretation as detailed below.

Major Point 1: Table 1 prominence and Level-1 criteria

> *These initial subdivisions were defined as "Subdivisions were primarily established in humans using high-resolution MRI and aligned with anatomical conventions from widely used atlases." Given the importance of other atlases in defining level-1 parcels Table 1 should have greater prominence. Right now, it is only shown in the methods and not present in the main text. The human subdivisions are not controversial, however the way this initial sub-division is defined inherently sets the stage for all subsequent analysis and naming given the "human-centric" approach. To understand the reliability of this, a clear set of criteria used to define the level-1 parcels and a more in-depth description should be provided for each species.*

We agree that Table 1 should be more prominent. It has been moved to the main text (Section 2.2) and substantially expanded. The revised table now includes five columns: (i) common hierarchical atlas (CHA) region name; (ii) definition and species-specific atlas coverage; (iii) spatial adjacency criteria stating the neighboring regions and their directions as defined during parcellation; (iv) geometric consistency (Geo. R), quantifying cross-species preservation of those spatial relationships; and (v) per-species nomenclature directness scores. Footnotes explain each metric and reference the full per-species pairwise adjacency analysis (Supplementary Table 2).

Regarding Level-1 criteria specifically, we have added a paragraph to the Methods (Section 4.2) clarifying the anatomical landmarks used to define the nine macro-regions. In humans, Level-1 boundaries follow established sulcal and gyral landmarks: the central sulcus separates frontal from parietal cortex, the Sylvian fissure delineates the temporal lobe and exposes the insula, the parieto-occipital sulcus marks the parietal-occipital boundary, and the cingulate sulcus defines the medial extent of cingulate cortex. For deep gray matter, the boundaries between basal ganglia and thalamic divisions follow clearly identifiable nuclear margins in high-resolution MRI. In nonhuman primates, homologous sulcal landmarks (central sulcus, lateral sulcus, parieto-occipital sulcus) were used where present, supplemented by atlas-defined boundaries from CIVM (rhesus) and Paxinos/MBM (marmoset). In rodents, where sulcal landmarks are largely absent, Level-1 boundaries were defined by registering the Allen Brain Atlas to the mouse MDT and grouping ABA regions into the nine macro-divisions based on literature-guided functional

correspondence (e.g., MOp and MOs to Frontal; SSp and SSs to Parietal; AUDp, AUDd, AUDpo, AUDv to Temporal). The rat Level-1 parcellation was derived by warping the mouse CHA. These criteria are now stated in the Methods and referenced in the main text alongside Table 1.

[Revised Methods Section 4.2 paragraph 2,

Revised Table 1 moved to Section 2.2,

new Supplementary Table 2]

Major Point 2: Containment validation and external validation

> *The authors attempt to validate their findings based on containment criteria of similarly named level-2 areas in the level-1 atlas. This is inherently circular and qualitative as they are validating different levels of the hierarchy based on whether they belong to one another across species. Yet level-1 subdivisions are not externally validated aside from atlas criteria which as mentioned in my first comment must be clearly specified for each species. Effectively, in each species they are using different levels of their own outputs to validate their outputs from another level of the atlas. I would propose an external validation criterion for the subdivisions made, not based solely on volumetry. This has recently been done quite extensively in the comparative neuroanatomy literature through connectivity (Xu et. al, 2020, Mars et. al, 2018), structure (Eichert et al, 2020) and transcriptomic features (Beauchamp et al. 2022).*
>
> *These approaches enable quantitative measures of how similar areas are across species based on features of the brain itself, and would provide sufficient validation to better ascertain the level of homogeneity claimed in each sub-division at either level-1 or level-2. While this may be impractical to do for all of the species used in the paper, cross species features and alignments are available for macaques (Xu et al., 2020, Mars et al. 2018, Warrington et al. 2020), and for mice (Beauchamp et al. 2022). I would ask the authors to use these resources to provide quantitative, non-volumetry validation of the proposed homologies.*
>
> *In short, the authors claim cross-species alignment of parcels based on a standardized anatomical framework. Yet the framework itself eschews actual quantitative alignment, and instead relies on the definitions of regions defined by prior atlases, and whether or not they belong to the intermediate parcel class defined by the authors through a circular containment criteria.*

(a) Containment clarification.

We thank the reviewer for the opportunity to clarify. Containment validation does not validate Level-2 against Level-1 within CHA. Rather, it evaluates CHA regions against external species-specific atlases (ABA for mouse, CIVM for rhesus, MBM for marmoset). Furthermore, containment does not assess homology; it quantifies boundary alignment loss incurred when synthesizing CHA from multiple, often inconsistent, species-specific atlases. Establishing cross-species homology at the fine resolution of species-specific atlases remains a fundamental challenge; CHA operates at a coarser scale where such homology can be more reasonably established. The containment metric asks a more modest but essential question: does CHA respect the boundaries of finer species-specific parcellations? For primates, where CHA was

synthesized from multiple sources (e.g., CIVM and INM for rhesus; MBM, Paxinos, and BMA for marmoset), validating against any single atlas provides a genuine external check. For mouse, where ABA was the sole source, 100% containment is expected by construction; this is a consistency check confirming no boundary violations were introduced. The lower scores in primates (94% ≥ 0.80 in rhesus; 79% ≥ 0.80 in marmoset) quantify the cost of reconciling inconsistent source atlases within a unified framework, analogous to the well-documented low Dice agreement among established human atlases (Figure 5a–b; Arslan et al., 2018; Bohland et al., 2009; Lawrence et al., 2021). As in humans, where no single parcellation can be treated as ground truth, sub-unity containment in primates reflects boundary disagreements across atlases rather than misalignment in CHA. These values therefore serve as practical benchmarks for future refinement.

(b) External validation.

We have now added connectivity-based validation comparing mouse tracer connectivity (~1,197 anterograde injections; Oh et al., 2014) against independent marmoset tracer connectivity (143 retrograde injections; Majka et al., 2020), both mapped to CHA. Despite differences in tracing methodology (anterograde vs. retrograde), species, and over an order-of-magnitude difference in dataset scale, the two matrices show significant correspondence (Spearman $\rho = 0.61$, $p < 10^{-11}$; new Figure 6) with agreement increasing with tracer coverage (quartile mean agreement: Q1 = 0.76, Q4 = 0.84; Figure 6c, inset). Sorted per-connection agreement values are provided in Supplementary Table 3.

Importantly, the Dice validation against Neuroparc (Figure 5) includes all human atlases in the repository (Desikan, Destrieux, HarvardOxford, Hammersmith, Juelich, AICHAJoliot2015, and others), not only the two source atlases used for CHA construction (FreeSurferSeg, Brodmann). CHA is therefore being compared against atlases it was not derived from, which addresses the concern about circularity.

A more extensive validation across all four species using diffusion MRI (Venkadesh et al., 2025) provides complementary evidence, particularly for the four cortical CHA regions excluded from the tracer comparison due to absent marmoset injection coverage (anterior insula, posterior insula, anterior olfactory, piriform). In that analysis, the entorhinal-hippocampal projection ranked as the most efficient pathway in all species, and other top-ranked conserved paths included thalamo-occipital and precentral-premotor connections, reflecting preservation of sensorimotor and memory-related circuitry. In contrast, anterior insula showed the strongest human-specific efficiency gains (both as source and target), while olfactory and piriform pathways showed peak efficiency in marmoset, diminishing in higher primates. These divergent regions are precisely those excluded from the tracer comparison, making the two analyses complementary.

Consistent with the transcriptomic comparison of Beauchamp et al. (2022), who reported greater mouse-human similarity for sensorimotor than association cortical regions, sensorimotor CHA regions such as precentral, parietal, and occipital cortex showed the strongest cross-species connectivity correspondence, while association and integrative regions such as temporal and insular cortex showed the greatest divergence. These patterns are also broadly

consistent with human-macaque comparisons using functional connectivity (Xu et al., 2020) and white matter connectivity blueprints (Mars et al., 2018; Warrington et al., 2020), which similarly reported greatest conservation in sensorimotor systems and greatest divergence in association cortex. That four independent approaches — invasive tracer connectivity, transcriptomic correspondence, non-invasive imaging, and directed connectomics cost efficiency analysis — converge on the same pattern of sensorimotor conservation and association divergence provides convergent support for the biological validity of CHA's regional definitions. We have cited these resources in the revised manuscript.

[New Figure 6, new Section 2.4.4, revised Discussion 3.1, 3.3, new Supplementary Table 3]

Major Point 3: Parietal nomenclature for mouse

> *While aiming for a common atlas is an admirable goal, it would be nice for the authors to discuss the appropriateness of the approach in comparative neuroanatomy. A specific and concrete example of this is presented in the discussion, "the mouse PAR parcel corresponds primarily to its continuous somatosensory territory, whereas primates possess a subdivided parietal cortex comprising postcentral, superior, inferior, and precuneus regions."*
>
> *I would ask the authors to extend and further justify their choice to call this the parietal cortex in the mouse. While I understand the aim is to provide a human-centric naming convention, calling somatosensory cortex parietal cortex is inherently misleading. In humans the parietal cortex contains major association hubs where sensory integration and multimodal processing occur (Wei et al. 2024). Calling somatosensory cortex the parietal cortex in this case is misleading both structurally and functionally.*
>
> *I do not understand how such a mismatch would not lead to further confusion in cross-species nomenclature and translation. If a mouse derived result is localized to the somatosensory cortex, then why would I report it as in the parietal cortex defined by the authors? Doing so now implies that my results occur in an area where multisensory integration occurs; extending associative function to a part of the cortex based solely on nomenclature. This is the intrinsic problem with renaming animal regions in a human-centric fashion without validation across multiple methods. Overall, when performing cross-species analysis it may be more precise to leave an imprecise match out rather then enforcing a nomenclature which does not fit the region it is assigned as it will only cause more confusion. The authors do respect this in the Acumbens, so why not for other regions where no clear match may exist?*

We thank the reviewer for this thoughtful critique, and we appreciate the comparison to accumbens, which raises an important principle about when cross-species labels are appropriate.

We respectfully note that the homology between mouse somatosensory cortex and primate parietal cortex is not absent; rather, it is partial. Mouse primary somatosensory cortex (SSp) corresponds directly to primate postcentral cortex (PAR_Postcentral), which is one of several subdivisions within the primate parietal lobe. The challenge is that primate parietal cortex also encompasses association areas (superior parietal, inferior parietal, precuneus) that have no clear homologue in mouse. In the hierarchical structure of CHA, assigning mouse

somatosensory cortex directly to PAR_Postcentral at Level 2 without a corresponding Level 1 parietal division would break the hierarchy. We therefore define PAR in mouse as a parietal territory (we have revised the manuscript to avoid referring to it as a 'lobe' in mouse) anchored by the clear somatosensory-postcentral correspondence while acknowledging that primate parietal cortex extends beyond this.

This situation is not unique to parietal cortex. Mouse primary and secondary auditory areas are assigned to TEM_Superior, even though primate superior temporal cortex encompasses language and auditory association functions well beyond mouse auditory cortex. In both cases, CHA does not claim functional equivalence; it provides common coordinate labels that enable systematic investigation of where species match and where they diverge.

The tracer comparison (Figure 6; Supplementary Table 3) illustrates why retaining PAR is important. Among the best-agreeing connections between mouse and marmoset are FRO_Precentral to PAR (mouse rank 0.92, marmoset rank 0.95, agreement 0.97) and the reciprocal PAR to FRO_Precentral (mouse rank 0.97, marmoset rank 1.00, agreement 0.97), consistent with conserved sensorimotor circuitry. In contrast, connections from association regions to PAR, such as FRO_Prefrontal to PAR (mouse rank 0.21, marmoset rank 0.70, agreement 0.51) and TEM_Inferior to PAR (mouse rank 0.11, marmoset rank 0.64, agreement 0.47), are among the least agreeing: these connections are relatively weak in mouse but strong in marmoset, consistent with the reviewer's point that primate parietal cortex has expanded association functions absent in mouse. Excluding PAR from mouse would prevent precisely this kind of quantitative comparison. It is only by assigning a common label that we can formally identify which connections are conserved and which diverge, which is one of the primary utilities of the framework.

We note that the Allen Brain Atlas itself uses the term 'parietal' for mouse cortex, designating posterior parietal association areas (PTLp) situated between somatosensory and visual cortex. ABA thus implicitly recognizes a parietal territory in mouse, with somatosensory cortex as its anterior division. Our framework makes this explicit at the macro level while deliberately excluding PTLp from CHA PAR because its homology to primate parietal association cortex is too uncertain, consistent with the principle of caution the reviewer advocates.

To address the practical concern about nomenclature confusion, we have added parenthetical species-specific identifiers to the mouse atlas labels where the CHA name differs from standard mouse terminology: PAR(SSp/SSs), TEM_Superior(AUD), TEM_Inferior(ECT), and FRO_Prefrontal(PL/ILA/ORB). This preserves the hierarchical structure and cross-species coordinate system while ensuring that mouse researchers immediately recognize the species-specific identity of each region.

{Revised Section 3.6, paragraph 2; rodent labels updated in release v1.1}

Major Point 4: Clarity of volumetric comparisons

> *The authors claim, "whereas rhesus macaques exhibited a relatively greater investment in temporal regions." Relative to which species? It has been previously shown that humans do have greater temporal innervation and expansion relative to other primates (Eichert et al. 2020).*

I would implore the authors to please write with the maximum amount for clarity which species is being compared to which in sentences such as this.

We thank the reviewer for this point and the reference to Eichert et al. (2020). We have revised all volumetric comparison statements to explicitly specify which species are being compared and to use MDT-level language throughout. We clarify that the original statement referred to volume fraction (proportion of total parcellated gray matter), not absolute size or connectivity strength. The rhesus MDT shows a higher superior temporal volume fraction (0.16) than the human MDT (0.08), because the disproportionate expansion of frontal and parietal cortices in the human MDT compresses the relative contribution of temporal regions. This does not contradict evidence of greater temporal connectivity or absolute expansion in humans (Eichert et al., 2020); it reflects the compositional nature of fractional volumetric data. Indeed, our follow-up study on directed connectomics across all four species using CHA (Venkadesh et al., 2025) found that superior temporal cortex showed significantly enhanced path efficiency in humans relative to other species, consistent with its role in language-related functions.

[Revised Results Section 2.3, revised Discussion 3.2, revised Figure 4 caption]

Major Point 5: Rat as non-core species

The rat was treated as a non-core species? Why? Multiple rat atlases including the Waxholm rat atlas exist and are freely available? https://ebrains.eu/data-tools-services/brain-atlases/rat-brain. It does not seem fair to simply assume that a mouse to rat registration should suffice when claiming homology across five different species, and an atlas for that species does exist.

We thank the reviewer for raising this concern and the reference to the Waxholm Space atlas. We have retained rat as a core species in the framework, with full mosaic views of the rat CHA added alongside the other four species in supplementary figures. Our rat MDT was constructed from 18 Wistar specimens using the SAMBA pipeline, representing the largest population-averaged rat template reported to date.

To validate the rat CHA, we performed containment analysis of Waxholm Space atlas regions (133 regions; Kleven et al., 2023) within CHA parcels (Figure 5d). Because the WHS is based on a single Sprague Dawley specimen while our MDT is Wistar, strain differences may contribute to boundary misalignment. To contextualize the containment results, we computed an independent inter-atlas baseline by evaluating WHS containment within the Duke Wistar atlas (54 regions; Johnson et al., 2021). Because CHA and the Duke Wistar atlas differ substantially in parcel number and size, comparing raw containment scores would be confounded by the tendency for larger parcels to achieve higher containment by chance. To address this, we revised the containment metric across all species to incorporate volume-weighting, ensuring that the comparison between CHA and the inter-atlas baseline reflects genuine boundary alignment rather than differences in parcel granularity (see revised Methods, Section 4.3.3). Under this adjusted metric, CHA containment (28% of volume at score ≥ 0.80) was comparable to inter-atlas agreement (31% of volume ≥ 0.80), indicating that boundary correspondence is limited by the available rat atlas infrastructure rather than by CHA itself. For comparison, marmoset achieved 79% and rhesus 94% of volume ≥ 0.80, reflecting the greater maturity of

atlas resources in those species. We have discussed these considerations in the revised manuscript (Section 2.4.3, Section 3.6, revised Figure 5d)

{Result Section 2.4.3 paragraph 3

Discussion Section 3.6, paragraph 3

Methods Section 4.3.3

Revised Figure 5d}

Major Point 6: Template novelty

> *The author's claim that a significant contribution of their work is the construction of templates used in their study. While building templates for their specific study population of rodents and macaques is likely helpful to their site-specific scanner, there are well established and widely used volumetric macaque/marmoset templates (https://prime-re.github.io/templates_and_atlases), and mouse templates (https://imaging.org.au/AMBMC/ https://www.science.org/doi/10.1126/sciadv.adq8089). Furthermore, upon looking at the details of the acquisition, number of animals and the source of the mice, is the mouse atlas the Duke mouse brain atlas? It appears to be the same IACUC approval, and the same data. In sum, given that a very similar mouse MDT exists, and the general availability of MDTs available the emphasis on the authors MDTs as novel findings are generally overstated.*

We thank the reviewer for this observation. We have revised the manuscript to clarify that our contribution is not the templates per se but the cross-species integration and hierarchical parcellation built upon them. Templates were selected or constructed according to consistent criteria: population-averaged minimal deformation construction, high-resolution isotropic acquisition, standardized orientation with the origin at the anterior commissure, and open-access licensing. Where existing community templates met these criteria, we adopted them directly (ICBM2009a for human, Brain/MINDS MBM v3 with AC-centered reorientation for marmoset). Where they did not, we constructed new MDTs: the mouse MDT uses Duke CIVM data (the reviewer is correct that these are the same specimens) reoriented to standardized horizontal alignment. For rhesus macaque, the NIMH Macaque Template (NMT v2; Seidlitz et al., 2018; Jung et al., 2021) provides a well-constructed population average from 31 specimens, but its native coordinate system places the origin far from the anterior commissure and does not follow AC-PC alignment. We therefore constructed a new rhesus MDT from PRIMatE Data Exchange scans with the origin at AC and AC-PC alignment applied during template construction.

Critically, all templates were then processed through a uniform 3D U-Net segmentation pipeline, ensuring consistent tissue classification regardless of template source. We have softened language throughout to reflect that the primary contribution is the hierarchical parcellation framework and its demonstrated utility, not the templates themselves.

[Revised Discussion Section 3.5]

Minor Point 1: Sagittal and coronal views

> *Visualization of some figures is confusing and incomplete to provide full context for the regions being shown. This is most evident in figure 3. Specifically, we are only shown axial views. This precludes the ability to assess the depth of medial structures such as the precuneus and cingulate. These views are key to actually understanding their position in the cortex.*

We agree that medial structures require additional visualization beyond axial views. We note that Figure 3 already provides coronal views for deep gray matter structures (basal ganglia, thalamus, hypothalamus). To address the reviewer's concern about medial cortical structures, we have added a supplementary figure (Supplementary Figure 6) showing 3D renderings of medial structures across all four species in both sagittal and posterior coronal views, displaying precuneus, medial temporal cortex, hippocampus, medial occipital cortex, anterior cingulate, and posterior cingulate. These views allow direct assessment of spatial positioning and cross-species differences. For example, the dorsomedial-to-ventromedial repositioning of posterior cingulate (CIN_Posterior) from rodent to primate, which underlies the lowest pairwise geometric consistency in the framework ($R = 0.36$; Figure 7a), is clearly visible in these views.

[New supplementary figure 6]

Minor Point 2: U-Net training and validation

> *The U-net results look fantastic and after going to the cited paper I am likely to use this tool myself. However, in the manuscript it would serve to explicitly state that it has been previously trained and validated in these species.*

We appreciate the reviewer’s positive assessment of the U-Net segmentation results. Tissue segmentation used a cascaded cross-species training procedure described in Yeh (2023), with separate hierarchies for primate and rodent lineages. Within each lineage, the species with the largest brain served as the initial model: human to rhesus to marmoset for primates, and rat to mouse for rodents. At each step, the leader model's segmentation was applied to the next species, manually inspected and corrected, and then used to train the follower model. This approach leverages superior signal quality in larger brains while ensuring accuracy through manual verification. We have added these details to the revised Methods.

[Methods Section 4.2 paragraph 1]

Minor Point 3: Containment definition placement

> *The definition of containment score comes far too late in the paper. It would serve the readability of the paper to explain it clearly in the main text instead of just the online methods.*

We agree, and have moved the containment definition earlier, introducing it in the Results section where containment validation is first discussed.

[Revised Section 2.4.3]

Minor Point 4: Rotation perturbation contextualization

> *Similarly, the rotation results are shown without any methodological contextualization. While I understand the ordering format of the paper, it would be good for this analysis to be partially explained while presenting the results.*

We have added a brief explanation of the rotation perturbation analysis in the Results section. The purpose is to demonstrate sensitivity of the containment metric: systematic degradation under rotation confirms that high containment scores reflect genuine anatomical alignment rather than chance overlap.

[Revised Section 2.4.3, revised Figure 5 caption]

Final Comments

> *The authors have taken an ambitious approach to provide a tool to harmonize atlas nomenclature for cross-species translation. While methodologically sound, further contextualization, and precision is needed to describe their findings. This includes exercising greater caution to prevent misnomers harmful to translation that create more confusion than they solve. To this end I would ask for full criteria for each species brought into the main-text, not relegated to the methods. I would also ask for validation of these parcellations in at least macaques and mice based on more than volumetric composition. I would also ask the authors to take into account the resources already available to the comparative neuroanatomy community, and specifically to expound on how their hierarchical atlas solves a problem not settled by using a precise species-specific atlas aligned through quantitative methods across-species?*

We appreciate the reviewer's constructive summary and the recognition that the approach is methodologically sound. We have addressed each point: (i) Table 1 with full criteria for each species is now in the main text, with spatial adjacency criteria, geometric consistency scores, and nomenclature directness scores; (ii) connectivity-based validation using invasive tracer data (new Figure 6) and directed connectomics (Venkadesh et al., 2025) now validates parcellations beyond volumetry, with convergent support from transcriptomic, functional connectivity, and white matter blueprint analyses; (iii) the Discussion now explicitly addresses how CHA complements species-specific atlases by providing a translational scaffold that enables formal cross-species comparison. CHA does not replace species-specific atlases or their nomenclature; it provides the missing cross-species coordinate system that links them, as demonstrated by the tracer validation and the directed connectomics application.

Code Availability

> *The code ran fine and the notebook for figures was well organized.*

We thank the reviewer for taking the time to run the code and for the positive assessment of its organization. All new analysis code (tracer validation, geometric consistency, homology confidence index) has been added to the Zenodo archive.

Response to Reviewer #2

Utility and new insights

This paper introduces a number of brain templates/atlases based on minimal deformation approaches for different species in an attempt to create a common space for cross-species analysis of developmental motifs, and other comparative approaches. It then uses these atlases to compare relative volumetric data across for some for some coarse brain areas. All data is provided to the scientific in a free and open format.

Cross-species comparison is an important approach for (developmental) neuroscience, and the creation of a common brain species could facilitate such approach. However, the work presented here, while solid in its underlying methodology, does not completely convince that this is the way to go. This could partially be a matter of presentation and argumentation, but the example brain areas that are compared across species do not seem to yield any new insights, which reduces the potential value of the 'framework' the authors introduce. The core idea of a common hierarchical framework is valid and interesting, but the current paper could use some work to reach its full potential and make it more appealing for the broad readership of Nature Communications.

The cross-species comparison heavily relies on the parcellations of the different species' brains and the assumption of homology. The former is optimized through the MDT approach and a U-Net segmentation for a first coarse segmentation. More refined (yet still relatively coarse) segmentation was then performed based on existing atlases and previously published work on anatomical homologies. In this step, parcels are kept constant as much as possible across species to facilitate comparison. This means that for cross-species comparisons, the parcellations reproduce existing parcels and known homologies which seems to make the discovery of new homologies unlikely. In the validation of the new 'Common Hierarchical Atlas' (CHA), the authors do show that their atlas is more similar to existing atlases than the existing atlases are to each other, meaning it summarizes existing knowledge well. Little or no metrics are provided about the U-Net based segmentation. In the methods section, the authors do refer to an entire preprint on this method by the senior author of this paper that provides such information, but some of this should be included in the current paper.

We thank the reviewer for recognizing the solid underlying methodology, the validity of the core idea, and the open data format. We agree that the original manuscript focused on volumetric comparisons, which primarily confirmed known patterns. The revision substantially strengthens the demonstration of utility through two new additions.

First, the cross-species tracer validation (new Figure 6) demonstrates that CHA enables formal comparison of invasive tracer connectivity across species, comparing ~1,197 mouse anterograde injections against 143 marmoset retrograde injections mapped to CHA (Spearman $\rho = 0.61$, $p < 10^{-11}$). This systematically identifies where rodent and primate connectivity architecture agrees and where it diverges, which directly informs the design of future invasive experiments: where mouse-marmoset correspondence is strong, primate tracing experiments need not be repeated; where they disagree, CHA pinpoints which regions most need primate-specific data.

Second, a follow-up directed connectomics study (Venkadesh et al., 2025) uses CHA to reveal both conserved memory circuits (entorhinal-hippocampal projection ranked most efficient in all four species) and lineage-specific differences (anterior insula efficiency peaking in humans, olfactory efficiency peaking in marmosets) across mouse, marmoset, rhesus macaque, and human. This analysis would not be possible without the cross-species coordinate system that CHA provides. We have added a new discussion section "Utility of the common atlas framework" to explain these points in greater detail.

We have also added U-Net segmentation details in methods as requested.

[New Figure 6, new Results Section 2.4.4, new Discussion Section 3.4, Methods Section 4.2, paragraph 1]

Multivariate volumetric analysis

> *The creation of the MDTs for each species and the subsequent segmentation seem fine. In the second objective, demonstrating how this information can be used for cross-species comparisons, the paper remains somewhat underwhelming. The authors investigate volumetric composition across species. Parcel volumes are normalized against brain size and relative contributions of parcels to total brain size are compared across species. While this is fine as a first approach, it does not really present a thorough cross-species analysis. What about looking at relative volumes within hierarchical parcels, or at combinations of volumes to investigate brain wide trajectories/patterns in a multivariate way? Would the increased contribution of one area not automatically lead to the decreased of other areas? This part of the paper should be expanded to yield more meaningful insights.*
>
> *The result of relatively large contribution of frontoparietal regions in primates and the olfactory system in rodents is not unexpected. Is there any insight the new common hierarchical framework can give us that wasn't already possible with previous atlases?*

The reviewer raises an important point about compositional constraints in volumetric data (i.e., that increased contribution of one area necessarily decreases others). We acknowledge that the volumetric analysis primarily confirms known patterns and have revised the text to present these as descriptive properties of the MDTs rather than novel findings. The more compelling demonstration of CHA's utility comes from the new connectivity-based analyses, which provide insights not achievable with species-specific atlases alone: the formal cross-species tracer comparison (Figure 6) and our follow-up directed connectomics study (Venkadesh et al., 2025). Multivariate volumetric approaches within and across hierarchical levels are a valuable direction for future work and are noted in the revised Discussion.

[New Figure 6, revised Results Sections 2.3 and 2.4.4, revised Discussion Sections 3.2 and 3.4]

Dice validation implication

> *What is the implication of the validation of the common atlas showing higher cross-atlas dice similarity with other atlases than the other atlases among themselves? Since the CHA*

parcellation was based on information from all these atlases it makes intuitive sense that it would be more similar to them than atlases that were not based on each other's information. Maybe I'm missing something, but this should be clarified. What is the implication of Fig 5a right panel? It suggests something Weber-Fechner law-like in the sense that regions with low dice coefficients to begin with have smaller differences between CHA and other atlas similarities. Are there outliers regions from the general trend? What does that mean?

We thank the reviewer for raising this point. We clarify that CHA was constructed using only two human source atlases (FreeSurferSeg and Brodmann areas), while the Neuroparc repository includes multiple additional atlases not used in CHA construction (Desikan, Destrieux, HarvardOxford, Hammersmith, Juelich, AICHAJoliot2015, and others). CHA is therefore being compared against atlases it was not derived from, which addresses the concern that higher similarity is expected by construction.

We frame the Dice validation result as non-inferiority: given the well-documented inconsistencies across established human brain atlases (Arslan et al., 2018; Bohland et al., 2009; Lawrence et al., 2021), no single parcellation can serve as ground truth for cortical boundary definitions. We now provide a Wilcoxon signed-rank test (median $\Delta = +0.125$, one-sided $p = 1.22 \times 10^{-4}$) confirming CHA performs at least as well as established inter-atlas benchmarks. That CHA median Dice consistently met or exceeded the existing inter-atlas agreement is noted as an encouraging but secondary observation.

Regarding the Weber-Fechner observation: the pattern in the right panel of Figure 5a reflects that regions with low baseline Dice (i.e., where all atlases disagree) have limited room for CHA to show improvement, whereas regions with moderate baseline Dice show larger ΔMedian values. This is consistent with the interpretation that CHA performs best where established atlases provide reasonable but imperfect correspondence, and adds little where atlases fundamentally disagree on boundary definitions. No individual region is a strong outlier from this trend. We have clarified this interpretation in the revised text.

[Revised Section 2.4.1, revised Figure 5 caption]

Discussion depth

The Discussion section summarizes the paper but does not provide a thorough discussion of the potential, limitation, and implications of the new atlas. While some are mentioned, they are mostly repetitions of what was said earlier in the paper. I think that if the value of the framework can be highlighted by additional cross-species analysis that yield new insight, the discussion can be expanded accordingly.

In its current form, the common hierarchical atlas framework presents an interesting approach, with solid underlying methodology for which the demonstration of added value and potential would require some additional work. I believe that if the authors can revise the paper in such a way, it would be a valuable resource for the field of comparative neuroimaging. In its current form it leaves such demonstration to others which strongly reduces the work's impact.

We appreciate the reviewer's assessment that the core idea is valid and interesting, and that the methodology is solid. We have substantially expanded the Discussion to include: (i) convergent validation across modalities (tracer, transcriptomic, imaging, directed connectomics); (ii) the per-region homology confidence index including its limitations and where future data would be most informative; (iii) broader perspective on applications including AI segmentation pipelines and transcriptomic integration; and (iv) demonstration of CHA's utility through the formal tracer comparison and the directed connectomics application.

[Substantially revised Discussion; new sections 3.3 & 3.4]

Code availability

> *I have looked at the code, but I have not reviewed it line-by-line. The code organization looks solid, with ipython notebooks to regenerate the manuscript figures, python code to generate the analyses, and proper documentation. I can only commend the authors for making this available in such an organized format.*

We sincerely thank the reviewer for taking the time to examine the code and for the positive assessment of its organization. All new analysis code (tracer validation, geometric consistency, homology confidence index) has been added to the Zenodo archive in the same organized format.

Response to Reviewer #3

> *This manuscript presents a novel hierarchical atlas for cortical and subcortical gray-matter regions across rodents (mouse, rat), nonhuman primates (marmoset, rhesus macaque), and humans. The authors construct population-averaged minimal deformation templates (MDTs) for each species to minimize anatomical variability and then apply a 3D U-Net for tissue segmentation (Level 0: GM, WM, DGM, CBL, CSF). They define Level 1 (nine macro-regions, e.g., frontal, parietal) and Level 2 (21–26 subdivisions) using landmark-guided boundaries and literature-based homologies. Validation includes Dice similarity against species-specific atlases and cross-scale containment scores. Volumetric analyses reveal species-specific scaling, such as expanded associative cortices in humans and limbic emphasis in rodents. The atlas is freely available and aims to support comparative neuroscience, connectomics, and translational studies.*
>
> *This work represents an important contribution to comparative neuroanatomy and addresses a key gap in cross-species brain mapping by integrating data from multiple species into a unified framework. However, there are several unclear points and methodological limitations that require major revision before the manuscript can be considered for acceptance.*

We thank the reviewer for recognizing this work as an important contribution that addresses a key gap in cross-species brain mapping. We have addressed all unclear points and methodological limitations as detailed below.

Methodological novelty

> *While the framework is useful, it lacks clear methodological novelty beyond the integration of existing tools (e.g., ANTs for MDT construction and U-Net–based segmentation). As noted in the Introduction, several initiatives have already harmonized atlases within primates; extending this approach to rodents is valuable, but the contribution would be more compelling if it incorporated novel alignment or parcellation strategies.*

We appreciate the reviewer's recognition that the framework is useful and that extending harmonization to rodents is valuable. We agree that the individual tools employed (ANTs, U-Net) are established methods, and we do not claim novelty in those components. Rather the contribution lies in the integration itself, the validation approaches developed to assess it, and the cross-species applications it enables.

Existing atlases were developed independently within single species using different template-construction methods and parcellation criteria, making it difficult to determine whether observed cross-species similarities or differences reflect true biology or methodological artifact. Consistency across the entire pipeline, from template construction through tissue segmentation to parcellation, is itself the methodological contribution: it is what allows regions and connections to be compared on equal footing across species. Within this consistent framework, we show that cross-species correspondence follows a structured gradient (sensorimotor conservation, association divergence), a pattern reported across independent modalities but

here captured within a single parcellation that enables direct region-to-region quantitative comparison rather than qualitative assumptions of homology (see Discussion, Section 3.1).

We introduce the first quantitatively validated cross-species parcellation with per-region homology confidence across rodents, non-human and human primates. It required reconciling inconsistent species-specific atlases across evolutionarily distant species (rodents through humans), establishing literature-guided homologies where direct anatomical correspondence is ambiguous, and defining spatial adjacency criteria that hold across all four species. No prior resource provides a unified parcellation spanning rodents, nonhuman primates, and humans within a single hierarchical framework.

The validation framework is itself a methodological contribution. We developed a composite per-region homology confidence index integrating three independent criteria (nomenclature directness, cross-species geometric consistency of regional positioning, and connectivity correspondence from independent invasive tracer data), providing the first quantitative assessment of confidence in cross-species regional assignments. The volume-weighted containment validation, by benchmarking CHA against species-specific atlases and an independent inter-atlas baseline in rat, also reveals the state of atlas infrastructure across species: the gradient from rat (28% of volume ≥ 0.80, matching a 31% inter-atlas baseline) to marmoset (79%) to rhesus (94%) quantifies where atlas resources are mature and where they remain limiting, information that is useful to the field independent of CHA itself. The cross-species tracer comparison, mapping mouse anterograde and marmoset retrograde data into CHA space despite differences in methodology, species, and dataset scale, represents a novel application of the framework that would not be possible without it. A follow-up study (Venkadesh et al., 2025) further demonstrates that CHA enables directed connectomics across four species by transferring mouse tracer-derived projection polarity through the common parcellation, an application that depends entirely on one-to-one regional correspondence across species.

Together, the parcellation, validation framework, and demonstrated utility constitute contributions that go beyond the assembly of existing tools. We have clarified this in the revised Introduction and Discussion (Section 3.5).

[Revised Introduction (paragraphs 3 and 4), new Results sections 2.4.4 and 2.4.5; Discussion sections 3.1, 3.3, 3.4 and 3.5]

Coarse parcellation

> *Level-2 parcellation includes only 21–26 regions, which is relatively coarse compared with existing fine-grained atlases (e.g., the Allen Brain Atlas with 372 regions). Although exclusions such as the claustrum, substantia nigra, and subthalamic nucleus are justified by MRI limitations, they reduce whole-brain coverage. In addition, the parietal cortex and caudoputamen remain undivided in rodents, which may oversimplify species-specific organization—particularly in smaller brains, where finer anatomical distinctions are critical.*

We thank the reviewer for this comment. The coarse parcellation is by design. It represents the resolution at which cross-species homology can be reasonably established across rodents,

primates, and humans. Finer parcellations are available within species-specific atlases; CHA provides the translational scaffold linking these finer definitions across species. As noted by the reviewer, parietal cortex and caudoputamen remain undivided in rodents due to the absence of clear anatomical boundaries at MRI resolution (the Allen Brain Atlas likewise treats caudoputamen as a single complex).

We note that retaining coarse regions does not prevent meaningful cross-species analysis; rather, it enables it. For example, the undivided mouse PAR parcel corresponds primarily to somatosensory cortex, whereas primate parietal cortex encompasses additional association areas. The cross-species tracer comparison (Figure 6; Supplementary Table 3) reveals that sensorimotor connections to PAR are among the best-conserved between mouse and marmoset (e.g., FRO_Precentral to PAR: mouse rank 0.92, marmoset rank 0.95), while association connections to PAR are among the most divergent (e.g., FRO_Prefrontal to PAR: mouse rank 0.21, marmoset rank 0.70). The coarse label thus serves as a starting point for quantifying where species converge and diverge, a major utility of the proposed parcellation.

We also wish to note that CHA does not replace existing species-specific resources; rather, it provides the missing translational layer that links them. Species-specific atlases remain the appropriate tool for within-species analyses at fine resolution. CHA operates at a coarser level where cross-species homology can be reasonably established, offering a coordinate system through which findings from one species can be formally related to another. By harmonizing parcellations across multiple species within a single framework, applying uniform tissue segmentation, and validating regional definitions against independent connectivity data, CHA complements the precision of species-specific atlases with the cross-species consistency that translational neuroscience requires.

Higher-resolution parcellations, especially at the subnuclear level in limbic and thalamic systems, are noted as a promising direction for future work in the revised Discussion.

[Revised Discussion Sections 3.5 and 3.6, new Figure 6, new Supplementary Table 3]

Male-only rodent data

> *Notably, mouse and rat cohorts are male-only, which may bias volumetric scaling analyses given known sex differences in brain structure.*

We acknowledge this limitation. The mouse MDT was constructed from male C57BL/6J mice, and the rat MDT from male Wistar rats. We have added a note in the Discussion acknowledging this sex limitation and noting that extending the framework to include both sexes is an important direction for future work.

[Revised Discussion 3.6]

Homology confidence

> *Although the atlas is literature-guided, some cross-species mappings (e.g., mouse ectorhinal cortex as a homologue of inferior temporal cortex) rely primarily on functional analogies rather*

than strict anatomical homology, introducing potential subjectivity. Providing confidence scores for each homology assignment or explicitly discussing uncertainty would improve transparency.

We thank the reviewer for this excellent suggestion, which prompted us to develop a quantitative per-region homology confidence assessment. We now provide a composite homology confidence index for each CHA region based on three independent criteria: (i) nomenclature directness, scoring how explicitly established species-specific atlases support each regional assignment (0–3 scale per species); (ii) geometric consistency, quantified as the mean resultant length of centroid direction vectors across species (Mardia & Jupp, 2009), measuring whether each region occupies a consistent spatial position relative to all other regions; and (iii) cross-species connectivity correspondence, derived from the new mouse–marmoset tracer comparison (new Figure 6), measuring per-region agreement in connectivity architecture between independently acquired invasive tracer datasets.

The combined index (new Figure 7b) ranges from 0.82 to 0.98 across CHA regions. Regions with the highest confidence (>0.95) have explicit nomenclature in all species-specific atlases and strong geometric consistency (e.g., hippocampus, amygdala, thalamus, piriform cortex). Regions with the lowest confidence (0.82–0.85) are those where at least one species relies on functional analogy, such as mouse ectorhinal cortex mapped to TEM_Inferior. We now explicitly mark the nature of each assignment in the revised Table 1, which also reports per-region geometric consistency scores alongside the spatial adjacency criteria used to define each boundary. Pairwise geometric consistency is presented in Figure 7a, where the lowest values reflect known neuroanatomical reorganization rather than parcellation error (e.g., CIN_Posterior–PAR, $R = 0.36$, reflecting the shift of retrosplenial cortex from the dorsal surface in rodents to the medial wall in primates).

We acknowledge the reviewer's specific example: the mapping of mouse ectorhinal cortex to CHA TEM_Inferior relies on functional rather than strict anatomical criteria. As noted in Table 1, ectorhinal cortex lacks the laminar complexity and cortical expansion of primate inferior temporal cortex, but shares connectivity properties including input from ventral visual areas and functional responses sensitive to object-level features (Nishio et al., 2018; Glickfeld & Olsen, 2017). The homology confidence index assigns this region a combined score of 0.82, the lowest in the atlas, transparently reflecting this uncertainty.

Four cortical regions (anterior insula, posterior insula, anterior olfactory, piriform) could not be evaluated on the connectivity criterion due to insufficient marmoset injection coverage, and all subcortical regions fall outside the scope of the corticocortical marmoset tracer dataset. These are explicitly flagged in Figure 7b. Their inclusion in the index based on the remaining criteria (nomenclature and geometric consistency) yields scores of 0.86–0.98, with the missing connectivity evaluation transparently indicated.

[New Figures 6 and 7, revised Table 1,

new Supplementary Table 2,

new Supplementary Table 3,

new Methods 4.3.4 and 4.3.5

new Results 2.4.4 and 2.4.5]

Human validation and cross-species atlas comparisons

> *Human validation relies primarily on Neuroparc atlases; comparisons with functional parcellations (e.g., Schaefer atlas) would strengthen the claims. Containment scores are substantially lower in marmosets (49% ≥ 0.95), which is attributed to atlas resolution, but this discrepancy warrants deeper analysis of atlas inconsistencies. For nonhuman primates, additional validation using functional connectivity benchmarks or comparisons with other cross-species atlases (e.g., the Chimpanzee Brainnetome) would be valuable. More generally, homology confidence should be quantified on a per-region basis.*

We thank the reviewer for the comment. Per-region homology confidence is now quantified through the composite index described above (Figure 7). Regarding the Neuroparc comparison, we clarify that this repository includes multiple independent atlases beyond our CHA source atlases (please see response to Reviewer 2, Dice validation), providing genuine external benchmarks.

In the revision, we updated the containment metric to incorporate volume-weighting, ensuring that comparisons are not confounded by differences in parcel granularity between target and reference atlases (see Methods, Section 4.3.3). This adjustment was necessitated by the addition of a rat inter-atlas baseline comparison, in which CHA and the Duke Wistar atlas differ substantially in parcel number and size. Under the revised metric, marmoset containment stands at 79% of volume ≥ 0.80 and 35% ≥ 0.95. To contextualize these values, the rat inter-atlas baseline (WHS regions evaluated within Duke Wistar parcels, both external to CHA) achieved only 31% of volume ≥ 0.80, establishing that even independently constructed species-specific atlases show limited boundary agreement. CHA rat containment (28% ≥ 0.80) matched this baseline, while rhesus achieved 94% ≥ 0.80. The gradient from rat to marmoset to rhesus reflects the maturity of atlas resources available in each species rather than a limitation of CHA itself. Where marmoset containment falls below unity, this reflects deliberate synthesis decisions in which we prioritized cross-species coherence over strict boundary preservation from any single atlas, as well as the relatively smaller number of MBM regions (121) compared to CIVM (220). The containment framework makes these decisions transparent and quantifiable.

The lower scores in marmoset quantify the cost of reconciling inconsistent source atlases within a unified framework, analogous to the well-documented low Dice agreement among established human atlases (Figure 5a–b; Arslan et al., 2018; Bohland et al., 2009; Lawrence et al., 2021). As in humans, where no single parcellation can be treated as ground truth, sub-unity containment reflects boundary disagreements across atlases rather than misalignment in CHA. These values therefore serve as practical benchmarks for future refinement.

We have substantially expanded validation in the revision through cross-atlas Dice benchmarking against established human parcellations (with statistical tests), cross-species geometric consistency of regional positioning, cross-species comparison of independent mouse and marmoset invasive tracer connectivity (new Figure 6), and cited convergent evidence from

functional connectivity (Xu et al., 2020), white matter blueprints (Mars et al., 2018; Warrington et al., 2020), and transcriptomic analyses (Beauchamp et al., 2022).

Our follow-up study (Venkadesh et al., 2025) provides additional validation by applying CHA to construct directed connectomes across all four species, integrating mouse tracer-derived projection polarity with species-specific diffusion MRI tractography. That analysis recovered canonical conserved circuits (the entorhinal-hippocampal core memory projection ranked as the most efficient pathway in all species) and identified biologically interpretable lineage-specific efficiency differences (anterior insula peaking in humans consistent with multimodal integration, inferior temporal outflows peaking in rhesus consistent with ventral visual stream specialization, olfactory pathways peaking in marmosets consistent with chemosensory communication). These results provide indirect functional validation of CHA's regional definitions through the biological coherence of the efficiency patterns they produce. Direct functional validation using parcellations such as the Schaefer atlas or cross-species resources such as the Chimpanzee Brainnetome would be a valuable complement, particularly as CHA advances to finer parcellation levels where functional boundaries become more informative. We note these as directions for future work.

[Revised Results section 2.4.1, new sections 2.4.4 and 2.4.5. New Figure 6, new Figure 7, Methods section 4.3.3, new Discussion section 3.3 and revised 3.6]

Statistical tests

> *No statistical tests are reported to support claims of similarity or superiority (e.g., in Figure 5, where the common atlas is described as providing superior or equivalent overlap). In mouse data, some regions show high similarity, but without statistical testing it is unclear whether this exceeds expectations based on literature-guided segmentation alone. Likewise, volumetric comparisons (e.g., "marmosets exhibited comparatively higher fractions…") are based on single MDT values per species; mapping regions to individual subject spaces and performing statistical analyses (e.g., ANOVA or permutation tests) would enable more robust inter-species comparisons.*

We thank the reviewer for this helpful comment.

Dice statistical tests: We now report a Wilcoxon signed-rank test comparing per-region median Dice similarity between "Common vs. Others" and "Others vs. Others." CHA achieved higher median Dice in 13 of 13 regions (median $\Delta = +0.125$, one-sided Wilcoxon signed-rank $p = 1.22 \times 10^{-4}$). We interpret this in a non-inferiority framework: given the well-documented inconsistencies across established human brain atlases (Arslan et al., 2018; Bohland et al., 2009; Lawrence et al., 2021), no single parcellation can serve as ground truth for cortical boundary definitions. The relevant question is therefore whether CHA introduces additional inconsistency beyond this baseline, and the results indicate that it does not. That CHA median Dice meets or exceeds inter-atlas agreement is noted as an encouraging, but secondary, observation.

Mouse ABA comparison: For the mouse ABA comparison (Fig. 5c), CHA mouse was deliberately constructed to follow human-centric nomenclature. Where ABA and CHA share direct correspondences (thalamus, hypothalamus, hippocampus, olfactory areas), Dice similarity is high, confirming spatial alignment. Where Dice is lower (e.g., the broad ABA isocortex parcel), this reflects intentional reorganization of mouse cortical territory into human-comparable divisions (e.g., grouping ABA auditory areas under TEM_Superior, visual areas under OCC_Lateral and OCC_Medial). This departure from ABA's mouse-centric organization is the core contribution of CHA for mouse: providing a translational coordinate system that maps mouse cortical regions into a framework directly comparable with primates and humans. Statistical comparison of CHA against ABA is therefore not appropriate, as the two atlases serve different purposes.

Volumetric statistics: The reviewer raises an important point regarding individual-level variability. The volumetric comparisons are derived from population-averaged minimal deformation templates (MDTs), which are constructed to minimize individual anatomical variability and provide a representative species-level anatomy. Accordingly, the reported volumetric fractions reflect central tendencies of regional organization at the species level rather than measurements from individual subjects. Formal statistical comparisons across individuals would require propagating CHA labels into individual subject spaces and explicitly modeling within-species variability in parcellation boundaries. While this is an important direction for future work, it is beyond the scope of the present resource-focused study. Importantly, our conclusions regarding cross-species proportional differences are descriptive properties of the MDTs and do not rely on inferential claims about individual variability. We have revised the manuscript to present volumetric comparisons explicitly as descriptive characteristics of the population-averaged templates (e.g., "the human MDT showed the largest proportional prefrontal volume" rather than "humans showed").

[Revised Section 2.4.1, revised Section 2.3, revised Figure 4 and 5 captions]

Detailed Questions and Requests for Clarification

Rat data inconsistencies

> *Rat data inconsistencies: The Introduction mentions mouse and rat data, but Figures 1 and 2 only show mouse results. Please clarify this inconsistency. How was the accuracy of rat atlas warping validated? Quantitative metrics (e.g., Dice similarity after warping) should be reported.*

We thank the reviewer for noting this inconsistency. In the revision, we have elevated rat to a core species in the framework, with full mosaic views of the rat CHA added to supplementary figures alongside the other four species. Our rat MDT was constructed from 18 Wistar specimens using the SAMBA pipeline, representing the largest population-averaged rat template reported to date.

To validate the rat CHA quantitatively, we performed containment analysis of Waxholm Space atlas regions (133 regions; Kleven et al., 2023) within CHA parcels (revised Figure 5d). Because

the WHS is based on a single Sprague Dawley specimen while our MDT is Wistar, strain differences may contribute to boundary misalignment. To contextualize the results, we computed an independent inter-atlas baseline by evaluating WHS containment within the Duke Wistar atlas (54 regions; Johnson et al., 2021). Containment scores were volume-weighted to ensure the comparison is not confounded by differences in parcel granularity between the two target atlases (see Methods, Section 4.3.3). Under this adjusted metric, CHA containment (28% of volume ≥ 0.80) was comparable to the inter-atlas baseline (31% of volume ≥ 0.80), indicating that boundary correspondence in rat is limited by the available atlas infrastructure rather than by CHA itself. For comparison, marmoset achieved 79% and rhesus 94% of volume ≥ 0.80. We have discussed these considerations in the revised manuscript (Section 2.4.3, Section 3.6, revised Figure 5d).

[Discussion 3.6 paragraph 3; Methods 4.3.3; new supplementary figure 5]

3D U-Net details

> *3D U-Net details: Please clarify the source and training procedure of the 3D U-Net model. If the network was trained on species-specific atlases (as implied by reference 39), this may bias segmentation toward those atlases and compromise independent validation. How was Level-0 segmentation accuracy assessed (e.g., Dice against manual labels)?*

We thank the reviewer for requesting clarification. The 3D U-Net performs Level-0 tissue classification (gray matter, white matter, deep gray matter, cerebellum, CSF) and is not trained on species-specific atlas parcellations. Training followed a cascaded cross-species procedure (Yeh, 2023) with separate hierarchies for primate and rodent lineages. Within each lineage, the species with the largest brain and superior signal quality served as the initial model: human to rhesus to marmoset for primates, and rat to mouse for rodents. At each transfer step, the leader model's tissue segmentation was applied to the next species, manually inspected and corrected, and then used to train the follower model. Because the U-Net classifies tissue compartments rather than anatomical regions, there is no circularity between Level-0 segmentation and the Level-1/2 parcellation, which is defined independently using anatomical landmarks and literature-guided homologies. We have added these training details to the revised Methods.

[Methods Section 4.2, paragraph 1]

Level 1 and Level 2 segmentation

> *Level 1 and Level 2 segmentation: More detail is needed on how gray matter was subdivided into Level-1 regions (e.g., criteria for separating prefrontal from parietal cortex). For Level-2 regions, clarify whether delineation was manual, automated, or hybrid. While Table 1 is informative, it lacks quantitative criteria for boundary definition.*

We thank the reviewer for this comment. Table 1 has been substantially expanded to address this concern. For each CHA region, the revised table now documents: spatial adjacency criteria (neighboring regions and their directions as defined during parcellation), geometric consistency

(Geo. R) quantifying cross-species preservation of those relationships, and expanded coverage descriptions integrating species-specific atlas labels and delineation method. CHA boundaries were derived from established species-specific atlases (ABA, CIVM, MBM, Paxinos, FreeSurfer/Brodmann) rather than de novo manual delineation. Where atlas labels did not directly correspond to CHA conventions (three regions in mouse: FRO_Prefrontal, TEM_Superior, TEM_Inferior), boundaries were landmark-guided using conserved spatial relationships and published functional evidence. The per-region homology confidence index (new Figure 7b) provides a composite quantitative assessment integrating nomenclature directness, geometric consistency, and cross-species connectivity correspondence, enabling readers to evaluate the strength of evidence for each regional assignment.

[Revised Table 1, new Figure 7]

Figure 4: Volume fraction definition

> *Figure 4 (Volumetric composition): Please define "volume fraction" explicitly and describe how it was calculated. Normalization to total brain volume per species is appropriate, but without individual-level data or statistical testing, comparisons based on single values are underpowered.*

Volume fraction is defined as the proportion of each region's volume relative to the total parcellated gray matter volume within each species MDT. We have added this definition to the revised Figure 4 caption. Because these measurements are derived from population-averaged minimal deformation templates rather than individual subjects, they reflect species-level descriptive properties and do not capture individual variability. As noted above, volumetric comparisons are presented as descriptive properties of the MDTs and do not rely on inferential claims.

[Revised Figure 4 caption]

Figure 5: Definitions, statistical tests, and spatial alignment

> *Figure 5 clarifications: Please define what constitutes "common" versus "non-common" atlases. No statistical tests are presented to support claims of superior or equivalent performance. How were species-specific atlases aligned to the common atlas for validation, and how was spatial misalignment quantified?*

Definitions: "Common vs. Others" refers to Dice similarity between each CHA region and its corresponding region in each Neuroparc atlas. "Others vs. Others" refers to Dice similarity computed among the Neuroparc atlases themselves, excluding CHA. This comparison tests whether CHA achieves at least equivalent spatial overlap with established atlases as those atlases achieve with each other. We have added these definitions to the revised Figure 5 caption.

Statistical tests: We now provide a Wilcoxon signed-rank test comparing per-region median Dice similarity between "Common vs. Others" and "Others vs. Others." CHA achieved higher

median Dice than inter-atlas agreement in 13 of 13 regions evaluated (median $\Delta = +0.125$, one-sided Wilcoxon signed-rank $p = 1.22 \times 10^{-4}$). Non-inferiority was also established at margins of 0.05 and 0.10 ($p = 1.22 \times 10^{-4}$ for both). We frame this result as non-inferiority rather than superiority: given the well-documented inconsistencies across established human brain atlases (Arslan et al., 2018; Bohland et al., 2009; Lawrence et al., 2021), no single parcellation can be treated as ground truth for cortical boundary definitions. The relevant question is therefore whether CHA introduces additional inconsistency beyond this baseline, and the results indicate that it does not. That CHA median Dice meets or exceeds inter-atlas agreement is noted as an encouraging, but secondary, observation.

Spatial alignment. For the human atlas, both CHA and the Neuroparc reference atlases are defined in ICBM152 standard space (Fonov et al., 2009), so no inter-atlas registration was required. The CHA human atlas (0.5 mm isotropic) was downsampled to 1 mm isotropic to match Neuroparc resolution prior to comparison.

For other species, species-specific atlases (ABA for mouse, CIVM for rhesus, MBM for marmoset) were registered to our MDT template spaces using nonlinear transformations prior to CHA construction. Because CHA was subsequently built within these same MDT spaces, the resulting CHA labels and the warped species-specific labels share the same coordinate system.

Quantifying spatial misalignment due solely to registration, independent of atlas boundary inconsistencies, is not straightforward for primates, as no single ground truth exists against which to measure registration accuracy. However, the mouse provides a direct test: because CHA mouse regions were derived exclusively from the Allen Brain Atlas, perfect containment (score = 1.0) (Supplementary Figure 7) confirms that the registration pipeline introduces no spatial misalignment between CHA and species-specific labels. For primates, where CHA was synthesized from multiple atlases that are themselves inconsistent with one another, containment scores below 1.0 reflect boundary disagreements across source atlases rather than registration artifacts. The rat provides additional context: an independent inter-atlas baseline (WHS regions evaluated within Duke Wistar parcels, both external to CHA) achieved only 31% of volume at containment ≥ 0.80, and CHA rat containment matched this baseline at 28%, establishing that boundary correspondence in rat is set by the available atlas infrastructure rather than by registration error or CHA limitations. The containment validation (Fig. 5d) transparently quantifies this boundary loss on a per-region basis using volume-weighted scores that account for differences in parcel granularity (see Methods, Section 4.3.3): rhesus (CIVM) achieved 94% of volume at containment ≥ 0.80, marmoset (MBM) achieved 79%, and rat (WHS) achieved 28% against a 31% inter-atlas baseline, with the gradient reflecting the maturity of atlas resources in each species.

We have revised the Figure 5 caption and Methods section to clarify these points.

[Revised Section 2.4.1, revised Figure 5 caption, revised Methods 4.3.1, 4.3.3]

Inter-rater reliability

> *Inter-rater reliability: For any manual boundary adjustments, inter-rater reliability should be assessed and reported. Providing volumetric values in tabular form would also improve reproducibility and reuse.*

CHA boundaries were derived from established species-specific atlases (ABA, CIVM, MBM, Paxinos, FreeSurfer/Brodmann) rather than de novo manual delineation, so inter-rater reliability assessment does not apply in the traditional sense. Where atlas inconsistencies required synthesis decisions, these followed documented criteria that are now quantified in the per-region homology confidence index (new Figure 7b): nomenclature directness scores how explicitly each source atlas supports the assignment, geometric consistency measures whether the resulting regions occupy consistent spatial positions across species, and connectivity correspondence provides independent functional validation. We have revised the manuscript to make these criteria explicit in the expanded Table 1.

[Revised Table 1, new Figure 7]

Figures: Abbreviations and color-scale thresholds

> *Figures: Improve figure clarity by expanding abbreviations in legends and providing quantitative color-scale thresholds (e.g., in Figure 4b).*

Thanks for this suggestion. Figure 4b includes a labeled quantitative color scale (*log(fraction_speciesA / fraction_speciesB*), range -1 to +1). We have ensured the caption explicitly states that positive values indicate greater fractional investment in species A.

Regarding abbreviations, key terms (tissue compartments, lobe-level regions, and species labels) are defined in the captions for Figures 2 and 3, where the parcellation hierarchy is introduced. Expanding all region abbreviations within every figure legend would substantially increase caption length without improving clarity for readers who have encountered the definitions in earlier figures. Instead, we have expanded Supplementary Table 1 into a comprehensive abbreviation reference covering all acronyms used throughout the manuscript, including atlas sources (ABA, CIVM, MBM, FSS, BA, INM, Paxinos), tissue classes (GM, WM, DGM, CBL, CSF), region shorthand (PreC, PreM, TemS, InsA, OlfA, etc.), template and methodological terms (MDT, ICBM2009a, AC-PC, U-Net), and imaging conventions (T1w, T2w, ROI). This table is referenced at first use in the main text and provides a single point of reference for all abbreviations.

[Revised Fig 4 caption, Revised supplementary table 1]

Discussion: Broader perspective

> *Discussion: The Discussion would benefit from a broader perspective on potential applications, such as use in AI-based segmentation pipelines or integration with transcriptomic (e.g., scRNA-seq) cell-type mapping efforts.*

We thank the reviewer for this excellent suggestion. We have added two paragraphs to the revised Discussion (Section 3.4) addressing these directions. First, we note that current deep learning approaches to brain segmentation are trained within single species and cannot produce cross-species homologous parcellations; CHA provides the first standardized ground truth that could support training of models that learn species-invariant anatomical features and generalize parcellation across species. Second, we discuss how transcriptomic efforts such as single-cell RNA sequencing have begun to characterize cell-type composition across species, but integrating these molecular data with macroscale anatomy requires a spatial framework defining regional correspondence; CHA provides this framework, enabling systematic comparison of cell-type composition within homologous divisions across species.

[new Discussion section 3.4]

REVIEWERS' COMMENTS

Reviewer #1 (Remarks to the Author):

The authors have significantly improved the clarity, readability, and scope of their manuscript through the revisions. I appreciate the move of Table 1 to the main text, even if doing so breaks the flow of the manuscript. By placing the primary findings (and their limitations) up front it is much clearer what they're CHA actually gives us as a community. Their clarification of the containment criteria and the rewrite of that section have substantially improved my understanding of what was done, and help limit the overclaiming I felt was present previously. I also especially appreciate the care taken to clarify areas that don't match: the paragraphs and parentheticals addressed to mouse researchers are doing real work in providing a clearer manuscript that avoids false equivalency when using this framework.

We thank the reviewer for the careful reading and for the generous assessment of the revised manuscript. We are glad that the rewritten containment section clarified what was done and reduced the earlier impression of overclaiming, and that the passages addressed to mouse researchers are helping the manuscript avoid false equivalency.

Overall, the authors have done well to address my prior concerns. On reading this draft, however, I do have one new major point. Could the authors describe how other researchers would integrate a new species, or a new atlas, into the existing framework? I think a common hierarchical atlas can be a useful tool for the community, but only if the broader non-human neuroimaging community can contribute alternate atlases for the species already included or add new species entirely. Can this be a tool that I can integrate my own data into? Could the authors discuss this, or — better still — implement it? The ability (or at least concrete plans) to extend this framework is, in my view, what determines the long-term impact of work of this kind.

We thank the reviewer for raising extensibility, which we agree determines the long-term utility of a common atlas. We have added a description of the integration procedure to the manuscript (Discussion, **"3.6. Integrating new atlases and species** ") and provided a standalone python implementation so that a reader can integrate their own data.

Integrating an alternate atlas for a species already represented in CHA is automated. The candidate atlas is aligned to the relevant species template (in DSI Studio or any registration tool), the containment fractions between its regions and the CHA regions are computed to form a containment table W, and each region is either assigned to its CHA region of maximum containment or, for atlas-level data such as a connectome, projected into CHA regions by the containment-weighted transformation $F_{CHA} = W^T\ F\ W$. We demonstrate this with the marmoset retrograde tracer (FLNe) connectome, which we project from Paxinos space into CHA and compare against the mouse tracer connectome (Fig. 6). The containment computation and the projection are provided as reusable functions and as a command-line tool (**`CHA_integrate.py`**) in the archived repository, with the marmoset integration as a worked example, so the same steps apply to a reader's own atlas and connectome.

Adding a new species rests on the established CHA cross-species homology mapping as a backbone, with an expert step whose size depends on phylogenetic distance. For a species close to one already represented (for example chimpanzee relative to human), regions can be initialized by nonlinear warping from the nearer species and then refined by manual delineation

from a neuroanatomist familiar with the major divisions of the new species being added, since anatomical nomenclature is not uniquely defined across species. We note the limitation of nonlinear warping here: the warp degrades with increasing anatomical divergence, so more distant species require correspondingly more manual delineation rather than reliance on the warp.

Finally, a few minor points remain:

1. In Table 1, CHA is not defined. I would recommend spelling it out either in the abbreviations list or at first use.

We have spelled out CHA (Common Hierarchical Atlas) at first use in the main text (**Introduction, paragraph 3**) and added the full term to the **Table 1 caption** so the table is self-contained.

2. The following line in the introduction is unclear: "Furthermore, most existing solutions are based on species-specific surfaces or templates and do not utilize population-averaged minimal deformation templates (MDTs), which are reference spaces created by iteratively averaging multiple individuals to minimize...
MDTs are not species-specific. I suspect the authors mean that most existing resources are based on a single subject, as opposed to a population-level construct like an MDT. The authors seem to clarify this in the next section, but mix terminology of templates and atlases (see next comment). In general, I think too much time is spent on the importance of MDTs. I'd prefer it be a more minor point since it's novelty is limited and they expand on the more novel aspects of their paper.

3. In the line: "In contrast, atlases for other species often rely on single-subject scans or templates not generated using comparable MDT frameworks, resulting in inconsistencies in spatial alignment and anatomical scaling," there is a meaningful distinction between templates and atlases that is being elided. Please be consistent and specific throughout the text about which is which — atlases label regions within a template space. Because this sentence directly follows the description of the human ICBM templates, it is unclear which object (atlas or template) is actually being referred to here.

These two comments concern the same paragraph, and we have rewritten it to address both (**Introduction, paragraph 2**). The contrast is now stated as single-subject versus population-averaged reference spaces rather than species-specific versus MDT, and we have removed the expanded MDT definition from the introduction, retaining only a brief mention and relying on the Methods for how the MDTs were constructed, in line with the reviewer's view that this is not a central novelty. We have also made the template-versus-atlas usage consistent: a template is the reference space and an atlas is the set of region labels defined within it. In the revised text, other-species atlases are described as defined within single-subject template spaces, with the alignment and scaling inconsistencies attributed to those spaces rather than to the atlases, and we have applied this terminology consistently throughout the manuscript.

Reviewer #2 (Remarks to the Author):

The authors have thoroughly improved the manuscript with this revision and for me addressed all comments and concerns. The addition of connectivity data and more thorough discussion have made this a much stronger paper.

We thank the reviewer for the positive assessment and are glad that the connectivity data and the expanded discussion strengthened the paper. We address the remaining comment below.

I only have one comment/question:
In the new Fig 7b, it's a bit confusing how the unavailability of connectivity scores affects the 'homology confidence'. If I understand the methods section correctly, the total score is now basically a division by 2 instead of 3? I'm not convinced this is the best way of accounting for non-available data. Would it not make more sense to score this as a neutral 0.5 and still divide by 3? Are the grey bars in Fig 7B now simply filling up to the average of nomenclature and geometric scores?

We thank the reviewer for this question, which prompted us to both clarify the scoring and revise the figure.

The reviewer is correct that the index is the mean over the available criteria, so for regions lacking connectivity it is the mean of the nomenclature and geometric scores (a division by two). We considered assigning a neutral 0.5 to the missing criterion and dividing by three, but chose not to, for two reasons.

First, the regions lacking connectivity data are the subcortical structures and four cortical regions without marmoset injection coverage (INS_Anterior, INS_Posterior, OLF_Anterior, OLF_Piriform). These include the most strongly conserved cross-species homologies (thalamus, basal ganglia, hippocampus, amygdala) and carry the highest nomenclature and geometric scores. Assigning 0.5 would treat a criterion that was never measured as if it provided only moderate support, when in fact it provides no information either way. A region with strong nomenclature and geometric agreement but no connectivity measurement would then be pulled down as though connectivity weakly argued against its homology, rather than being silent on it. Several of the most strongly conserved structures would fall below cortical regions with weaker agreement, reversing an ordering that the nomenclature and geometric criteria, and classical neuroanatomy, jointly support.

A second consideration is that 0.5 may not be neutral for the connectivity criterion in this dataset. The observed connectivity-correspondence scores cluster well above 0.5 (range 0.733 to 0.899), so the expected value for an unassessed region would be considerably higher than 0.5. A more representative substitute would be the average of the observed scores, and using it gives essentially the same result as the mean over the available criteria. We therefore retain the mean-over-available index, which makes no assumption about the missing criterion, and we now state this explicitly in the Methods and figure caption.

On the figure, the reviewer's confusion about the grey bars was well founded, and we have redesigned **Fig. 7b** to remove it. The previous stacked bars with a grey placeholder for the missing criterion made it unclear what the grey encoded relative to the score. The panel now shows the three criteria as separate bars on their native 0-1 scale, with the combined index marked by an open rectangle ending at its value (printed at right). Regions without connectivity data simply have no connectivity bar, so the index visibly reflects the two available criteria and there is no grey segment to interpret. The revised caption states that for these regions the index is the mean of the available criteria.

Reviewer #3 (Remarks to the Author):

The authors have thoroughly addressed all of the reviewer comments in the revised manuscript. The revisions are clear, comprehensive, and significantly improve the quality and presentation of the work. The responses provided are satisfactory, and the changes made demonstrate careful consideration of the feedback. I appreciate the effort invested in strengthening the manuscript. In its current form, I believe the manuscript is suitable for publication.

We thank the reviewer for careful reading across the revisions and for the recommendation to publish. We are glad the changes addressed the earlier comments and strengthened the manuscript.
